# Benchmarking unsupervised methods for inferring TCR specificity

**DOI:** 10.1093/nargab/lqaf150

**Published:** 2025-11-19

**Authors:** Charline Jouannet, Hélène Vantomme, Kenz Le Gouge, David Klatzmann, Encarnita Mariotti-Ferrandiz

**Affiliations:** Sorbonne Université, INSERM, Immunoregulation-Immunopathology-Immunotherapy (i3), 75005, Paris, France; AP-HP, Hôpital Pitié-Salpêtrière, Biotherapy UF, CIC-Paris Est, 75013, Paris, France; Sorbonne Université, INSERM, Immunoregulation-Immunopathology-Immunotherapy (i3), 75005, Paris, France; AP-HP, Hôpital Pitié-Salpêtrière, Biotherapy UF, CIC-Paris Est, 75013, Paris, France; Sorbonne Université, INSERM, Immunoregulation-Immunopathology-Immunotherapy (i3), 75005, Paris, France; Sorbonne Université, INSERM, Immunoregulation-Immunopathology-Immunotherapy (i3), 75005, Paris, France; AP-HP, Hôpital Pitié-Salpêtrière, Biotherapy UF, CIC-Paris Est, 75013, Paris, France; Sorbonne Université, INSERM, Immunoregulation-Immunopathology-Immunotherapy (i3), 75005, Paris, France; AP-HP, Hôpital Pitié-Salpêtrière, Biotherapy UF, CIC-Paris Est, 75013, Paris, France; Institut Universitaire de France, France

## Abstract

Identifying T-cell receptor (TCR) specificity is crucial for advancing the understanding of adaptive immunity. Despite the development of computational methods to infer TCR specificity, their clustering behavior has not been thoroughly compared. We addressed this by curating a unified database of 190 670 human TCRs with known specificities for 2313 epitopes across 121 organisms, combining data from IEDB, McPAS-TCR, and VDJdb. We asked whether widely used TCR clustering methods produce comparable results on the same high-confidence dataset. We hypothesized that shared assumptions about conserved CDR3 motifs would yield similar patterns, with differences reflecting algorithmic design. Nine methods for clustering TCRs based on similarity were benchmarked against this dataset. DeepTCR demonstrated the best retention, while ClusTCR, TCRMatch, and GLIPH2 excelled in cluster purity but had lower retention. GLIPH2, Levenshtein distance, Hamming distance, and clusTCR generated large clusters in contrast to TCRMatch and DeepTCR. Smaller, antigen-specific clusters were produced by GIANA and iSMART. DeepTCR was the most sensitive in capturing antigen-specific TCRs. We confirmed these observations using a larger dataset from 10X Genomics containing antigen-specific labeled TCRs as well non-labeled cells. This study offers a unified TCR database and a benchmark of specificity inference methods, guiding researchers in selecting appropriate tools.

## Graphical Abstract

**Graphical Abstract:**
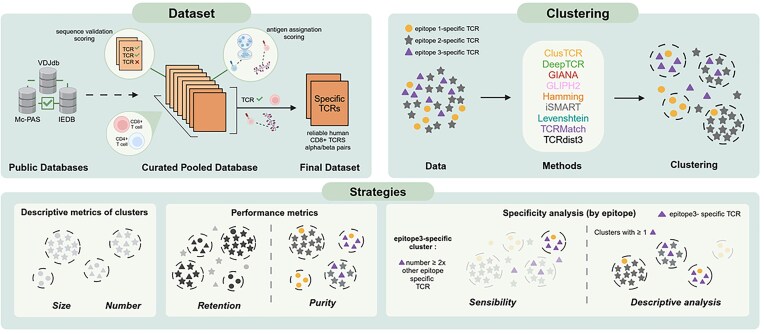

## Introduction

T cells are characterized by the expression on their surface of a unique antigen-specific receptor, called the T-cell receptor (TCR). The TCR is a heterodimer formed with two immunoglobulin superfamily chains, the alpha (TCRα) and the beta (TCRβ) chains in most of the T cells, independently generated through a recombinatorial mechanism between a collection of Variable (V), Diversity (D), and Junction (J) gene segments [1]. This heterodimer is the T-cell unit that recognizes antigen-derived peptides bound to major histocompatibility complex (MHC) [2]. These chains are generated by somatic rearrangements of various gene segments encoded, respectively, on the TRA (for the TCRα chain) and TRB (for the TCRβ chain) genomic loci. In humans, the TRA locus is composed of 54 Variable (TRAV), 61 Joining (TRAJ), and 1 Constant (TRAC) gene segments [3], whereas the TRB locus is composed of 50 TRBV, 13 TRBJ, 2 TRBC, and 2 additional Diversity (TRBD) gene segments. During thymopoiesis, the V(D)J recombination starts on the TRB locus: a TRBD gene segment first joins a TRBJ gene segment, followed by a TRBV gene segment joining the TRBD-TRBJ complex to form a TRBV-TRBD-TRBJ rearrangement. The TRBC gene segments is later added through splicing during transcription. The recombination process at TRA locus begins after successful TRB selection, once a functional TRB is expressed, and proceeds by direct TRAV-TRAJ gene segments joining. Allelic exclusion ensures the most cells express only one productively rearranged chain by locus. However, exceptions occur : dual-TCR expression is relatively common for TRA and rare for TRB (often reported up to ~30% dual-TRA and much lower frequencies for dual-TRB) [4, 5]. Finally, random nucleotide deletions and/or insertions happen over the recombination process. This recombination generates a highly variable region at the junction of the assembled gene segments called the complementarity determining region 3 (CDR3), which is in direct contact with the peptide. The T-cell specificity is defined by the reactivity of a given TCR to a given peptide, entailing physicochemical interactions between the peptide-MHC complex and the V(D)J region, which engage T cell on its activation. It is well established that T cells are cross-reactive through their unique TCR, therefore capable of recognizing multiple antigens [6]. Unravelling this antigen specificity is challenging due to the requirement of multicellular and molecular interactions. So far, TCR antigen specificity has been characterized based on preconceived *in vitro* or *in vivo* assays with well-established peptides, far beyond the universe of possible interactions [6]. As such, they are not powerful enough to generalize easy and accurate screening for TCR specificity.

With the advent of next-generation sequencing of the TCR and the concomitant development of computational tools, new approaches have been proposed to infer the TCR specificity based on sequence similarity and pattern identification. These tools are all on the ground foundation that TCRs recognizing identical peptides exhibit conserved motifs and sequences within their CDR3 regions. Several methods and tools for TCR specificity inference have been proposed. Among the most used, the Levenshtein distance (LD) [7, 8] and the Hamming distance (HD) [9, 10] as well as tools such as TCRMatch [11] and TCRdist3 [12] have been conceived based on sequence similarity measures. Other approaches also commonly used, such as clusTCR [13], iSMART [14], GIANA [15], GLIPH2 [16], and DeepTCR [17] are rather focused on motif identification and clustering.

Despite their availability, each method’s clustering behavior has not been analyzed using the same metrics and datasets, thus hindering the ability to compare their relative clustering behavior. A recent study by Hudson *et al.* compared the predictive performances of part of the abovementioned tools [18] using a partially curated version of the VDJdb containing pairs of TCR alpha (TRA) and beta (TRB) chain rearrangements annotated with their antigen specificity. Their results showed comparable performances between the tools, regardless of the model used behind for inference, with very sophisticated tools performing as well as a simple Hamming distance, for example. Moreover, the authors found that depending on the dataset (three different annotated TCR databases), the prediction performances were different. They suggested that these discrepancies could be due to different sequence preprocessing. Another possible reason is the degree of reliability of antigen-specificity annotations in these databases. Indeed, not all TCRs from these databases were unambiguously assessed for their antigen specificity, which could lead to multiple false positive annotations and therefore prediction.

As such, it remains to assess to what extent do widely used computational methods, which infer TCR specificity from sequence similarity, produce comparable clustering results when applied to the same high-confidence dataset. We hypothesized that, since these methods are all built on the premise that TCRs recognizing the same antigen share conserved motifs in their CDR3 regions, they should produce similar clustering patterns when applied to the same curated dataset. However, we cannot exclude that differences in algorithmic design, from simple distance metrics to motif-based approaches, embedding-based representations, and deep learning models, may nonetheless lead to measurable differences in clustering behavior, especially under noisy conditions.

Therefore, we propose here a refined benchmarking of the most used tools using a curated database that combines pairs of TCRs from three publicly available databases, VDJdb, IEDB, and Mc-PasTCR, together with unified scoring for antigen-specificity determination reliability.

## Materials and methods

### Curated and unified database generation

The dataset used in this analysis is based on a combination of three publicly available databases: Immune Epitope DataBase (IEDB) [19], VDJ database (VDJdb) [20], and the manually curated catalogue of pathology-associated TCR sequences (McPAS-TCR) [21], with the data being collected as of March 2023. Within the IEDB database, accessed via iedb.org, specific parameters were set: “Any Epitopes” in the Epitope field, “T Cell” and “Positive” outcome in the Assay field, “Human” as the host, “Any” in the Disease field, and “TCR alpha/beta” as the TCR type. To accommodate analyses based on MHC restriction types, this database was accessed twice with distinct settings for MHC restriction: “MHCI” to identify CD8+ T-cell related sequences and “MHCII” for those associated with CD4+ T cells. For both VDJdb and McPAS-TCR, filters were applied to select only data pertaining to human species. Notably, within these two databases, every sequence is documented with information on the cell subset. Subsequently, these three databases were combined into a unique dataset, undergoing extensive curation for uniformity of information. In cases where entries across the three databases shared identical information (same V-CDR3 amino acid-J for both TRA and TRB, same epitope, same organism, same PubMed ID, and same cell subset), only one instance was retained (486 sequences were involved). If there were missing information or discrepancies between the databases for the same source, each instance was preserved to leave the choice to the user.

For each TRA/TRB pair, a sequence reliability score, named *Verified_score* (VS), was established. The verification process of the sequence was primarily conducted using the IEDB, relying on its comprehensive curation strategy [19]. We assessed the concordance between the “calculated” and “curated” sequences by considering the start and end positions of the CDR3 sequences, as well as the delineation at the “C–F” border. For the other two databases, assignment of information was contingent upon the availability of both the alpha and beta CDR3 sequences. A score of 2 indicates that both TRA and TRB sequences are known and verified; a score of 1.1 is attributed when only the TRA sequence is present and verified, and a score of 1.2 when this is the case for the TRB sequence; finally a score of 0 is set when neither sequence is verified. Similarly, the method of antigen identification is used to assign a second score, *Antigen_identification_score* (AIS), allowing users to filter on this parameter as detailed in Supplementary Table S1.

**Figure 1.**
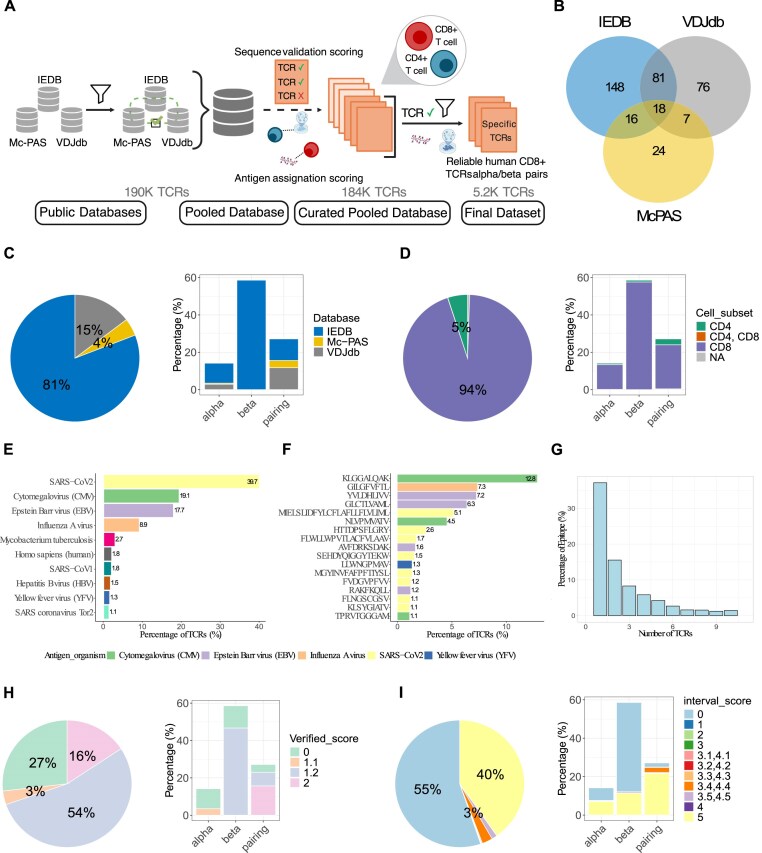
Overview of the pooled database composition. (**A**) Curation strategy: human TCRs extracted from three public databases, IEDB, VJDdb, and Mc-PAS, are combined into a large dataset. After applying several filters (see the “Materials and methods” section), the pooled database is filtered according to the cell type CD8+ T cells. (**B**) Venn diagram representing overlap of studies across the three public databases. (**C**) Database contribution: overall distribution of public databases in the final pooled database in the whole data (left panel), and their distribution across different chain types: alpha, beta, and alpha/beta pairings (right panel). (**D**) Cell type distribution: in the entire pooled database in the whole data (left panel) and across each chain type: alpha, beta, and alpha/beta pairings (right panel). (**E**) Predominant antigen organisms: barplot illustrates the most represented antigen organisms in the pooled database, with numbers indicating the percentage of TCRs binding to each organism. (**F**) Key epitopes: barplot displays the most represented epitopes in the pooled database, with numbers reflecting the percentage of TCRs binding to each epitope. (**G**) Epitope–TCR binding analysis: barplot showing a focus of the distribution of epitopes in the pooled database based on number of TCRs binding to them. For representation purposes, we cut the axes to 10 TCRs as it covers ∼80% of the data. Nevertheless, there are epitopes with >10 TCRs, one epitope being recognized by 18222 TCRs. (**H**) Verified score distribution across the entire pooled database (left panel) and within each chain type (right panel). (**I**) Antigen_identification_score (AIS) distribution in the entire pooled database (left panel) and within each chain type (right panel).

For the purpose of the benchmarking, we excluded sequences with unknown epitope annotation, as well as those with low annotation reliability, by keeping only entries with a VS of 2 and an AIS above 4.3. We then applied additional filters designed to ensure that the dataset reflected biologically meaningful and comparable TCR sequences across all tools: (i) selection of pairings with known V and J information, as V/J usage is directly incorporated into the similarity measures of several methods, (ii) exclusion of pairings with unknown epitope, (iii) selection of TCRs with CDR3s of 6–23 amino acids, consistent with CDR3 length distribution observed in large-scale studies [22–28], which captures the vast majority of productive, functional CDR3 sequences, and (iv) selection of epitopes that bind at least two TCRs defined as a unique CDR3α/CDR3β pair, to further improve cluster robustness and reduce noise from singletons. Moreover, TCRs originating from 10X genomics datasets were excluded from the combined database as these data were used as an independent validation set for subsequent analyses. The curated-resulting dataset from these filters comprises 5261 TCRs from CD8+ T cells and 192 TCRs from CD4+ T cells, all with known VJ gene segments and epitope information. The curation strategy for this pooled database is depicted in Fig. 1A and detailed in the Materials and Methods section.

### Antigen-specific TCR dataset

We retrieved a publicly available TCR dataset generated by 10x Genomics genomics from CD8+ T cells of four human donors with single-cell resolution on gene expression, expression of 11 surface proteins, and paired αß TCR sequences together with peptide–MHC binding, providing information on TCR specificity (CD8+ T cells of healthy donors sorted for dextramer positive cells: https://zenodo.org/records/6952657). Antigen specificity was assessed using 44 MHC class I dextramers (Immudex), each loaded with a distinct antigenic peptide derived from different viruses (CMV, EBV, Influenza virus, HTLV, HPV, and HIV) or from known cancer antigens. Moreover, six dextramer reagents carrying irrelevant peptides were added as negative controls. Each dextramer was labeled with a distinct nucleic acid barcode and a phycoerythrin fluorophore. In the following analysis, we focused on data from Donor 1. We filtered cells according to the following criteria: (i) selection of high-confidence productive cells, (ii) selection of cells with a maximum of three TCR chains (one TRB and up to two TRAs), (iii) if two TRAs were associated with the same TRB, only the TRA with the greatest read count was selected, and (iv) selection of CDR3 sequences between 6 and 22 amino acids in length. After filtering, 86 034 cells remained, corresponding to 48 954 unique CDR3 sequences. A CDR3 was considered bound to an MHC dextramer when his binding score exceeded a threshold defined by a UMI count >10, as recommended in the 10x Genomics application note.

Noise spike-in design: we constructed six subsets with increasing noise while keeping the annotated pool fixed; subset_1: only annotated TCRβ/CDR3β (*n* = 2876), subsets_2–6: random samples (without replacement of non-annotated TCRβ/CDR3β spiked into the annotated set to reach total sizes of 7613; 12 350; 17 087; 21 824; 26 561 sequences, respectively).

### Benchmarked tools and approaches

We benchmarked the performance and the clustering behavior of widely used approaches and tools selecting them based on the availability of open-source packages, scripts, or executable files, and ensuring representation across the broad spectrum of antigen-specificity inference approaches, including sequence similarity, motif identification, deep learning, and hierarchical clustering. This process yielded the following nine shortlisted methods: Levenshtein distance (LD) [7, 8], Hamming distance (HD) [9, 10], TCRMatch [11], TCRdist3 (v.0.2.2) [12], clusTCR (v.1.0.2) [13], iSMART [14], GIANA (v.4.1) [15], GLIPH2 [16], and DeepTCR (v.2.0) [17]. Note that only GLIPH2, DeepTCR, TCRdist3, and ClusTCR can integrate paired TRA and TRB as input. Hence, for LD, HD, TCRMatch, GIANA, and iSMART, TRA and TRB rearrangements were analyzed independently, whereas for TCRdist3, clusTCR, GLIPH2, and DeepTCR, both TRA and TRB rearrangements from paired TCRs were considered. All the methods, but TCRdist3, were run using their default parameters, as recommended by the respective authors, to ensure that we did not interfere with their original configurations. For TCRdist3, as no standardized clustering pipeline exists, we optimized the hierarchical clustering on the output distance matrix using the silhouette score. For DeepTCR, the option of optimization threshold for the hierarchical clustering was also chosen (already implemented in the original package). Additional information regarding each approach and the parameters used are detailed in the Supplementary data.

### Clustering behavior and similarity metrics

Assuming that the similarity of CDR3 amino acid sequences correlates with epitope specificity, as supported by evidence that higher similarity in the CDR3 region of TCRs increases the likelihood of shared epitope recognition [29], some metrics were set out to assess the effectiveness of the clustering approaches mentioned above, although, this task is not trivial, given the complexity of TCR–epitope interactions. These metrics, widely adopted in TCR clustering benchmarking studies [30, 31], include purity, retention, and sensitivity, which together offer a solid framework for evaluating the quality of the clusters formed. In details

Retention is defined as the ratio of the number of clustered TCR/CDR3 sequences to the total number of sequences in the dataset. As not all input sequences end up in the final clustering results, this measure represents the number of TCR/CDR3 sequences (*s*) that belong to any cluster (*c*). Retention relies only on the capacity of the approach or tool to cluster TCR/CDR3, with no implication on the impact of cross-reactivity. The calculation follows the formula:


\begin{eqnarray*}
\textrm{retention} = \ \frac{{\sum \left| {s\in c} \right|}}{{\sum \left| s \right|}}.
\end{eqnarray*}

Purity is defined as the fraction of TCR/CDR3 sequences within a single cluster targeting the same epitope. Purity is calculated as the sum of TCR/CDR3 (*s*) specific for the most represented epitope ($\gamma )$ within each cluster (*c*) divided by the total number of sequences in any cluster. The purity values range from 0 to 1, where 0 indicates non-pure clusters (all TCRs/CDR3s bind to different epitopes) and 1 indicates pure cluster (all TCRs/CDR3s bind to a same epitope). The calculation follows the formula:


\begin{eqnarray*}
\textrm{purity} = \ \frac{{\sum \left| {\gamma \left( {s\in c} \right) = \ {{\gamma }_{\mathrm{ max} \left( c \right)}}} \right|}}{{\sum \left| {s\in c} \right|}}.
\end{eqnarray*}

As opposed to retention, purity considers the information of antigen specificity, therefore it may be penalized in case of cross-reactivity. Purity thus penalizes polyspecific clusters, which are generally considered undesirable. However, as polyspecificity can also reflect true biological cross-reactivity [27], we interpret purity together with retention and epitope sensitivity to distinguish artefacts from genuine biological signals. For this reason, purity is interpreted alongside retention and epitope sensitivity to better disentangle artefacts from biologically relevant overlaps.

Based on the purity score, we further calculated

the percentage of clusters with a purity over 0.9, defined as the proportion of clusters in which the predominant epitope is the most represented (>90% of the cluster). For each cluster, individual purity is calculated as
  \begin{eqnarray*}
\textrm{purity}\ \left( {\textrm{cluster}} \right) = \ \frac{{\gamma \left( {s\in c} \right) = \ {{\gamma }_{\mathrm{ max} \left( c \right)}}}}{{s\in c}}.
\end{eqnarray*}

Then, the percentage of clusters with a purity >0.9 is determined as the number of clusters with purity >0.9 divided by the total number of clusters:


\begin{eqnarray*}
\mathrm{ perc}{_{\textrm{clusters}}} = \ \frac{{\sum \left| {c\left( {\textrm{purity} > 0.9} \right)} \right|}}{{\sum \left| c \right|}}.
\end{eqnarray*}

the percentage of TCRs/CDR3s in clusters with a purity over 0.9, defined as the fraction of sequences that belong to the clusters previously identified as having a purity >0.9.
  \begin{eqnarray*}
\mathrm{ perc}{_{\mathrm{ CDR3s}}} = \ \frac{{\sum [s\in {{c}_{\left( {\textrm{purity} > 0.9} \right)}}|}}{{\sum \left| {s\in c} \right|}}.
\end{eqnarray*}

Sensitivity is defined as, for a given epitope $\gamma $, the fraction of epitope-specific TCRs/CDR3s contained within epitope-specific clusters $( {{{c}_{\textrm{specific}}}} )$. Epitope-specific clusters are selected based on two criteria: a size >3 sequences and a number of epitope-specific sequences at least twice higher than the other sequences in the cluster. Then, the sensitivity for each epitope is calculated as


\begin{eqnarray*}
\textrm{sensitivity}\ \left( {\textrm{epitope}} \right) = \frac{{\sum \left| {\gamma \left( {s\in {{c}_{\textrm{specific}}}} \right)} \right|}}{{\sum \left| {\gamma \left( s \right)} \right|}}.
\end{eqnarray*}

Retention and purity, including the percentage of clusters with a purity >0.9 and the percentage of sequences/pairs belonging to these high-purity clusters (as previously described), as well as sensitivity were computed on the output of each method. First, these metrics were calculated on the global clustering outputs from the prefiltered curated database (filters mentioned above). Then, the analysis was extended to epitope-specific sequences. For this purpose, we selected six epitopes as detailed in the “Result” section, each represented at various extent in the input database.

### Tool robustness analysis under noise

Unless stated, sequences assigned to clusters of size ≥2 are considered clustered. Retention and purity metrics were computed as defined in the “Clustering behavior and similarity metrics” section. Beyond these, we report (i) persistence of annotated sequences. For subset_1, let A1 be annotated sequences that are clustered, for subsets  ∈2, …, 6 , let A be annotated sequences that are clustered; we compute, which assesses whether sequences clustered without noise remain clusterable after adding noise, without requiring cluster identity matching; (ii) distribution of non-annotated sequences by cluster size: among non-annotated clustered sequences, the percentage assigned to clusters of sizes 2–3, 4–10, 11–50, 51–100, and >100; and (iii) cluster composition by non-annotated sequences: for each cluster (size 2), the proportion of non-annotated sequences, with clusters binned into <10%, 10%–30%, 30%–60%, 60%–90%, 90%–99%, and 100%, reported as the percentage of clusters per bin (unweighted). All methods were executed three times on the same fixed subsets. Due to memory limits on our virtual machine, DeepTCR could not be executed on subsets 5–6; we did not impose an ad-hoc fixed threshold, to preserve comparability with runs where clustering is internally optimized. Reproducibility: The compositions of all six subsets were fixed after their initial generation and reused in every run. Therefore, any variability observed across repeats results exclusively from internal stochasticity or auto-optimization of the algorithms, rather than from resampling.

### Statistical analysis

All statistics analyses were performed using R software version 4.1. Comparative figures were produced using ggplot2 R package [32], networks were produced using Cytoscape software version 3.10.1 [33], and explicative schemas were created with biorender.com.

## Results

### Publically available TCRs are enriched for CD8 T-cell TCRs and viral antigen specificities

To assess the clustering behavior of antigen-specificity inference methods of αβ TCRs, we combined and curated three well-established public databases: IEDB [19], McPAS-TCR [21], and VDJdb [20] (Fig. 1A). These three databases were composed of TCRs, i.e. of unique CDR3a/CDR3b pairs, from overlapping studies (Fig. 1B) representing nearly 25% of the total number of TCRs (for the remaining 75%, the source is not indicated). Specifically, 18 studies were common across the three databases, 81 shared between IEDB and VDJdb, 16 between IEDB and McPAS-TCR, and 7 between VDJdb and McPAS-TCR.

IEDB contributes to 81% of the pooled database, followed by VDJdb at 15%, and McPAS-TCR at 4% (Fig. 1C, left). In detail, VDJdb and McPAS-TCR provide predominantly αβ TCR pairs (11.7% and 3.9%, respectively), some α-only chains (2.9% and 0.6%, respectively), but no β-only chains (Fig. 1C), while IEDB is mainly composed with β-only chains. In addition, 94% of the publicly available TCRs originates from CD8+ T cells (Fig. 1D). An overview of the most representative antigen organisms and epitopes for which the TCRs were annotated (>1%) and included in the pooled database is presented in Fig. 1E and F. The predominant organism is SARS-Cov2 (~40%), followed by the Cytomegalovirus (CMV; 19%) and Epstein–Barr virus (EBV; 18%) (Fig. 1E). The top three epitopes are KLGGALQAK (CMV), bound by 12.8% of TCRs, GILGFVFTL (Influenza), bound by 7.3%, and YVLDHLIVV (EBV), bound by 7.2% of the TCRs present in the curated pooled database. Figure 1G illustrates that most epitopes (>35%) are associated with only one TCR. These epitopes and their associated TCRs were excluded from further clustering analyses. Altogether, we assembled a pooled and unified database of 190 670 TCRs among which 184 137 TCRs recognize 2313 known epitopes from 121 organisms.

### Unified scoring reveals database heterogeneity and provides tool for informed TCR selection

The TCRs populating these databases were originally identified with different degrees of sequence reliability (verification of the original nucleotide and amino-acid sequences for each TRA and TRB rearrangement) and antigen-specificity validation (e.g. cell culture with peptide or full protein, tetramer/dextramer cell sorting, expansion in or association with disease). To overcome the heterogenous level of sequence reliability (assessed by the VS) and antigen-specificity reliability (assessed by the Antigen_identification_score (AIS)), we extended the scoring on sequence validation provided by IEDB and on antigen-specificity annotation provided by VDJdb to our pooled database (detailed in Dataset). Twenty-seven percent of the TCR entries was classified as unreliable (VS = 0, meaning that the sequences were not double checked). Moreover, there are 54% of reliable TRB sequences (VS = 1.2), much higher than the 3% of validated TRA sequences (VS = 1.1). Finally, while only 15% of the TRA/TRB pairs and 20% of TRB-only chains are unreliable, the proportion of unreliable sequences is higher for TRA-only chains (75%). Nevertheless, some TRA/TRB pairs feature unreliable TRA (VS = 1.2, 27%) (Fig. 1H). Regarding antigen-specificity validation, 55% had an AIS of 0, meaning that no information was available regarding the method used to assess the antigen specificity. Conversely, 40% of the sequences have an AIS of 5 (Fig. 1I, left panel). Focusing specifically on TRA sequences, we observed roughly equal proportions of sequences with no (48.1%) and high AIS (51.9%). In contrast, nearly 80% of TRB chain sequences are categorized as unreliable. Conversely, for the TRA/TRB pairings, the majority of sequences (~80%) exhibits a high AIS. Altogether, our unified scoring revealed the heterogeneity of the quality of the information available for the sequences stored in the databases and now offers an objective process to select the TCRs of interest and evaluate the clustering behavior of antigen-specificity inference tools (Fig. 1I, right panel).

### Most of the antigen-specificity inference methods form small clusters

Given the bias toward TCRs originating from CD8 T cells in the unified database, our benchmarking analysis was focused on those predominant TCRs, further narrowed down to only those with a VS = 2 and an AIS > 4.3. As such, the actual analyzed dataset was composed of 4779 unique TRA/TRB pairs, including 4103 unique CDR3a and 4292 unique CDR3b (8395 unique sequences). For the LD, HD, TCRMatch, iSMART, and GIANA methods, we analyzed only the CDR3 sequences, as per the method original design, and considered the two chains separately. In contrast, for the GLIPH2, clusTCR, DeepTCR, and TCRdist3 methods, we inputted the paired TCRa/TCRb sequences along with the required additional information in line with their original description (see details in Supplementary Table S2).

**Figure 2.**
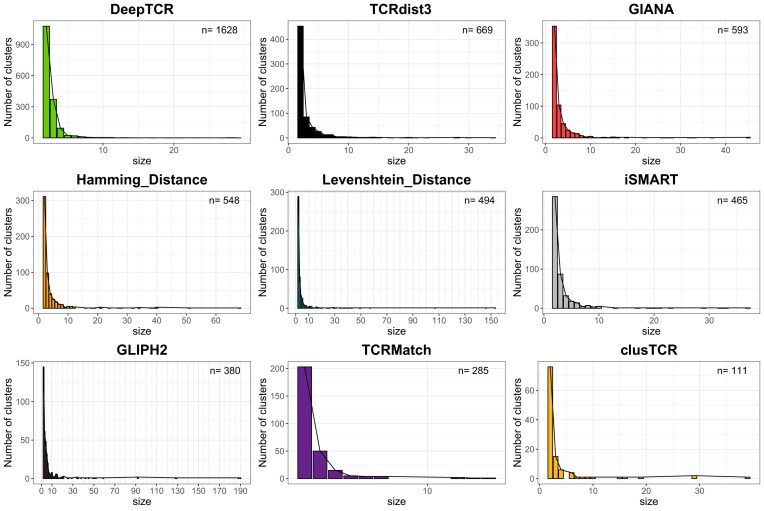
Comparative clustering analysis of the nine methods. Cluster size distribution for the nine methods, ordered by decreasing number of clusters.

By assessing the number of clusters formed and their sizes (Fig. 2 and Supplementary Table S2), we found that ClusTCR creates the least number of clusters (111), while DeepTCR creates the highest number (1628). The size distribution of the cluster is comparable across all the methods, with the majority (>70%) of the clusters being small (<4 sequences), suggesting a low similarity among sequences in the database. The size of the largest cluster varies from 14 (TCRMatch) to >150 (LD) CDR3/pairs, with TCRdist3, GIANA, iSMART, and clusTCR generating clusters no larger than around 40 CDR3/pairs. Interestingly, these differences can be observed despite a similar number of clusters (e.g. LD versus TCRdist3, GIANA, and iSMART), highlighting differences in the clustering parameters.

When we ran the clustering on the full database with mixed TCRa/CDR3a and TCRb/CDR3b, we found that LD, HD, TCRMatch, iSMART, and GIANA, originally not designed for TRA/TRB pairing input, formed pure clusters composed of either TCRa/CDR3a or TCRb/CDR3b, as illustrated by the network visualization (Supplementary Fig. S1A and B). Network visualization for clusTCR, GLIPH2, DeepTCR, and TCRdist3 are also available in Supplementary Fig. S1C.

### DeepTCR shows the highest retention and clusTCR and GLIPH2 the highest purity for TCR clustering

**Figure 3.**
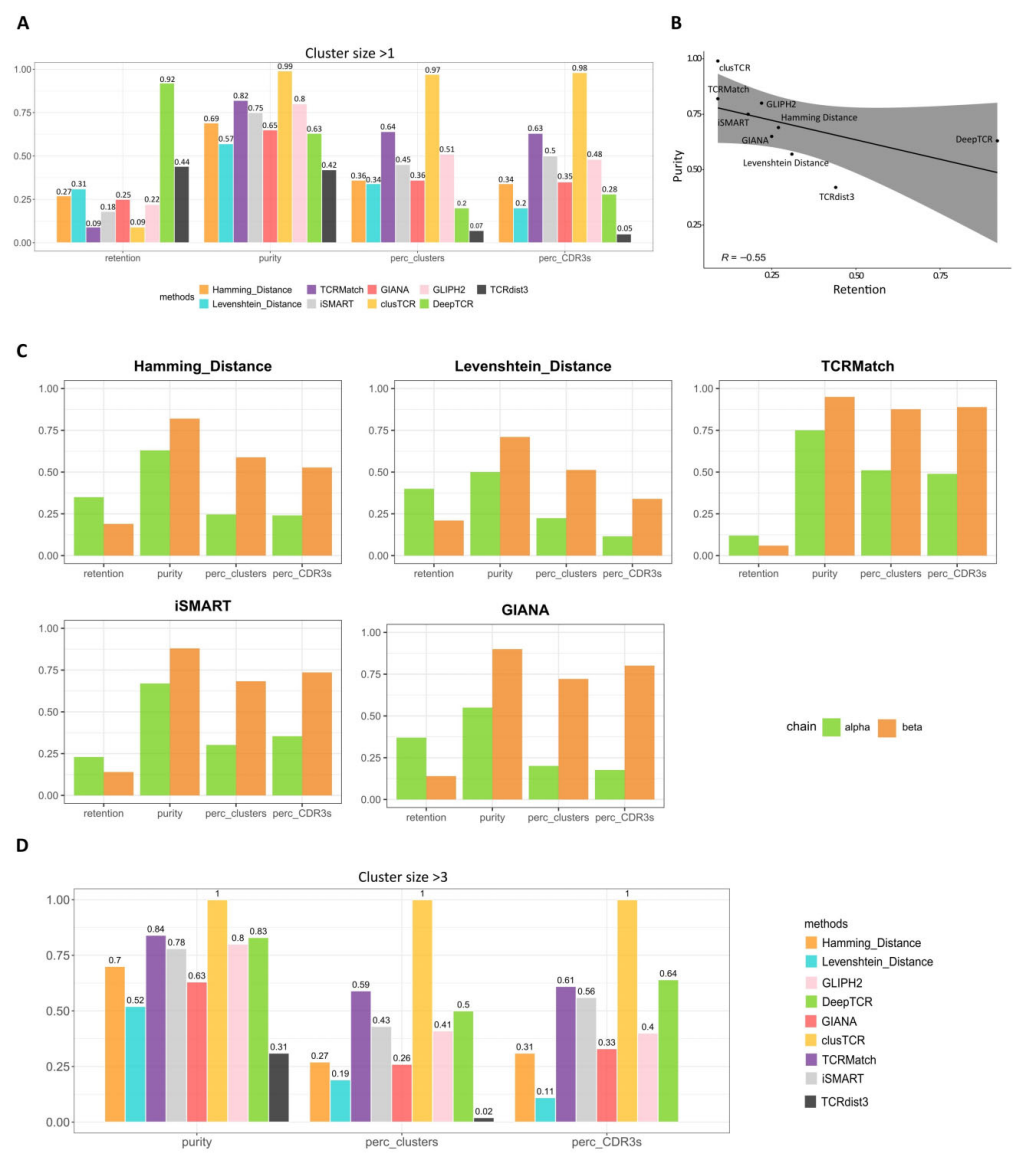
Evaluation of method clustering behavior. (**A**) Clustering behavior metrics: barplot showing different clustering behavior metrics for the nine methods, including retention, the purity, the percentage of clusters with a purity over 0.9, and the percentage of sequences within these clusters. All metrics are normalized between 0 and 1 (with the last two metrics needing multiplication by 100 to convert their values into percentages). (**B**) Correlation plot of the purity as a function of the retention. (**C**) Clustering outcomes when α and β chains are analyzed separately. Specific metrics as panel (A): retention, purity, percentage of clusters with a purity over 0.9, and percentage of sequences within these clusters for the HD, the LD, TCRMatch, iSMART, and GIANA, analyzed separately for alpha and beta chain clusters. (**D**) Same analysis as in panel (A) but with a focus on clusters with more than three sequences.

To compare how antigen specificity is captured by the clustering approaches, we computed the retention, as the number of sequences actually clustered out of the total number of input sequences, and the purity of the clusters, as the fraction of the sequences annotated with the most abundance antigen specificity covered among all clusters (see the “Materials and methods” section and Supplementary Fig. S2). Figure 3A summarizes the results for each metric across all methods and Supplementary Table S2 details the number of sequences clustered. LD, HD, GIANA, and iSMART showed similar low retention (0.25 ± 0.06) and intermediate purity scores (0.66 ± 0.09), whereas TCRMatch displayed a very low retention (0.09) and much higher purity (0.82). DeepTCR outperforms all the methods in terms of retention (0.92) and displayed an intermediate purity (0.63). clusTCR clusters the lowest number of sequences (retention = 0.09) with a purity of 0.99, similar to TCRMatch. GLIPH2 ranks in the third place regarding the purity (0.8) with a low retention to 0.22. Surprisingly, TCRdist3 stands out for balancing both metrics (retention = 0.44, purity = 0.42). In summary, we found a negative correlation between purity and retention (Fig. 3B), revealing subtleties driven by each method. These results suggest that TCR with close-enough sequences can recognize distinct epitopes, as supported by recent studies [27, 34, 35].

We further quantified the fraction of highly pure clusters (at least 90% of purity) and the sequences/pairs forming these clusters. Interestingly, 97% of clusTCR clusters are of at least 90% purity, capturing 98% of the sequences clustered. TCRMatch, GLIPH2, and iSMART showed intermediate scores (64%, 51%, and 45% clusters with at least 90% purity, respectively; and capturing 63%, 48%, and 50% of the sequences, respectively), followed by LD, HD, and DeepTCR (34%, 36%, and 20% of highly pure clusters capturing 34%, 20%, and 28% of the sequences, respectively). Conversely, TCRdist3 has the lowest score values (7% of 90% purity clusters; 5% of the pair). Of note, when the TRA and TRB chains were analyzed separately using HD, LD, TCRMatch, iSMART, and GIANA, we found that both the retention and the purity are lower for the TRA chain than the TRB chain (Fig. 3C). Given that all the sequences were initially from reliably annotated pairs of TCRs, this result suggests a differential contribution of each chain to antigen specificity, as proposed earlier [36].

However, as shown in Fig. 2, most of the methods generate predominantly small clusters made of two to three sequences, a bias that can influence the global purity score. We then computed the purity considering clusters composed by strictly more than three sequences or pairs (Fig. 3D). Purity remained consistent with previous results for most of the methods, except for LD, TCRdist3, and DeepTCR (0.52, 0.83, and 0.31, respectively). For LD and TCRdist3, while overall purity was comparable, we found a two-fold decrease in high purity cluster percentage (19% and 2%, respectively) and in the percentage of sequences in these clusters (11% and 2%, respectively), revealing that the purest clusters were those formed by at most three sequences, while larger clusters are formed with sequences with different specificities. For DeepTCR, the overall purity (0.83), the percentages of high purity clusters (50%), and of pairs in these clusters (64%) increased, suggesting that clusters smaller than three sequences mainly were heterologous. These results were further confirmed when considering clusters larger than 5 and 10 sequences (Supplementary Fig. S3). The number of clusters formed when considering clusters size >3, 5, or 10 sequences/pairs is shown in Supplementary Table S3.

Altogether, our results indicate major differences regarding sequence/pair retention depending on the methods, with DeepTCR being the most conservative and TCRMatch the least.

### Polyspecific TCRs are captured by most of the methods

**Figure 4.**
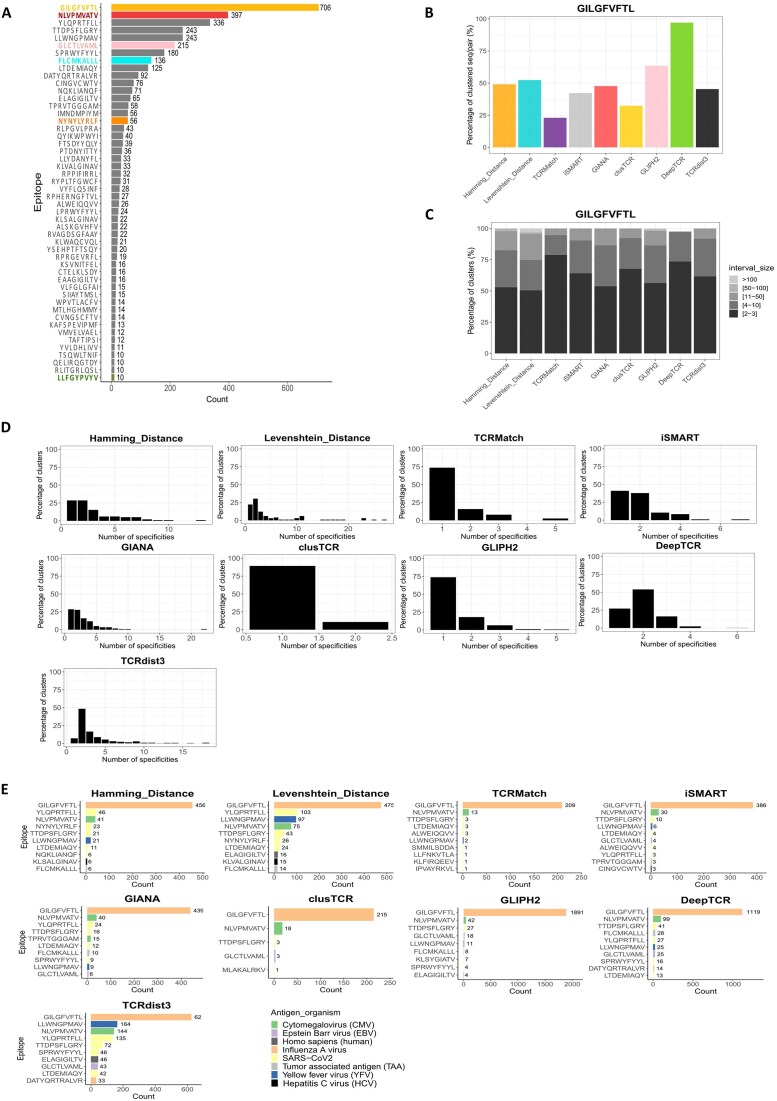
Analysis of method clustering behavior using specific epitopes. (**A**) Representation of top epitopes, most-represented epitopes in the input data, focusing only on those with a frequency >1%. Six epitopes were selected based on their frequency (both high and low) and are color-coded as follows: dark gold for GILGFVFTL, red for NLVPMVATV, pink for GLCTLVAML, blue for FLCMKALLL, orange for NYNYLYRLF, and olive for LLFGYPVYV. (**B**) Percentage of GILGFVFTL-specific-sequences/pairs clustered by each method. (**C**) Size interval distribution in GILGFVFTL-specific clusters for each method. Clusters are GILGFVFTL-specific, if they contain at least one CDR3/pair that recognizes GIL. (**D**) Specificity distribution in GILGFVFTL-specific clusters as a function of the number of specificities contained in each cluster, for each method. One specificity was chosen for polyspecific CDR3/pair. (**E**) Top additional epitopes in GILGFVFTL-specific clusters displaying the top 10 (or fewer) additional epitopes present in these clusters for each clustering method, colored by antigen organism. The numbers displayed on the barplot correspond to the total number of sequences specific to the different epitopes associated with the specific GILGFVFTL clusters.

We further quantified the numbers of specificities per cluster. More than 70% of the clusters formed by HD, LD, GIANA, iSMART, DeepTCR, and TCRdist3 assemble TCRs with at least two specificities, i.e. in polyspecific clusters, whereas TCRMatch, clusTCR, and GLIPH2 predominantly formed monospecific clusters (Supplementary Fig. S4). The distribution of the antigen-specificities in the database is far from being even. As such, some specificities are highly represented, such as GILGFVFTL (later named GIL) associated with 706 distinct TRA/TRB pairs, as compared to others relatively less represented, like LLFGYPVYV associated with only 10 pairs (Supplementary Table S4 and Fig. 4A). To evaluate the impact of such imbalance, we focused our analysis on the clusters formed by each method with at least one CDR3/pair specific to a given epitope (GIL). Consistent with the overall retention, DeepTCR clusters nearly 100% of the GIL-specific annotated pairs, while TCRMatch followed by clusTCR grouped <30% of the same pairs (Fig. 4B). HD, LD, GIANA, and iSMART retain ~50% of the sequences. GLIPH2, despite a moderate retention score, captures ~67% of GIL-specific pairs. Importantly, for all the methods, half or more of the GIL-specific clusters are formed by at most three sequences (Fig. 4C).

We then further analyzed the distribution of the other specificities present in the GIL-labeled clusters. TCRMatch, clusTCR, and GLIPH2 predominantly formed monospecific GIL-specific clusters whereas HD, LD, iSMART, GIANA, DeepTCR, and TCRdist3 favored polyspecific clusters (Fig. 4D). The distribution of the other specificities present in the GIL clusters was strikingly comparable between all the methods generating polyspecific clusters (Fig. 4E), with the epitopes most frequently identified among all nine methods being YLQPRTFLL, TTDPSFLGRY (from SARS-Cov2), NLVPMVATV (from CMV), and LLWNGPMAV (from Yellow Fever virus). The same trends were found when analyzing clusters labeled with a less-represented epitope in the database (FLCMKALLL; Supplementary Fig. S5). These observations suggest that the ability of the method to capture antigen-specific sequences, including clustering polyspecific TCRs, is independent of the abundance of the antigen-specific TCRs in the database.

### DeepTCR shows highest sensitivity in epitope-specific TCR clustering, while TCRMatch and TCRdist3 show the least

**Figure 5.**
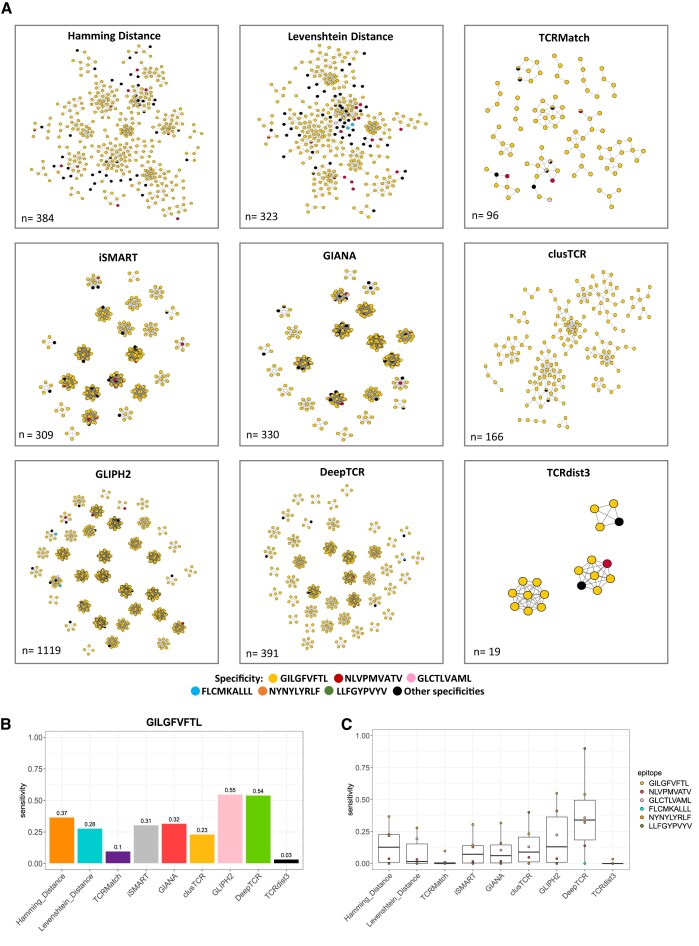
Focused analysis on GILGFVFTL-specific clusters and sensitivity. (**A**) Network visualization of the purest clusters representing clusters selected where GILGFVFTL is the predominant epitope, being at least twice as abundant as the second most used epitope in the cluster across each method. (**B**) Sensitivity of the GILGFVFTL epitope for each method. (**C**) Epitope wide-sensitivity comparison for the six selected epitopes across each method. Statistical significance was assessed using pairwise Wilcoxon tests between methods; no significant differences were detected.

We next selected more specific clusters based on two criteria: a size greater than three sequences and a number of epitope-specific sequences at least twice higher than the other sequences in the cluster. We further analyzed the sensitivity of each method selected for the GIL epitope focusing on clusters where the GIL sequences/pairs predominated (Fig. 5A and Supplementary Fig. S6), as detailed in the “Materials and methods” section (Fig. 5B). GLIPH2 followed by DeepTCR exhibited the highest sensitivity, indicating their effectiveness in capturing GIL-specific pairs. Conversely, TCRdist3 showed the least sensitivity. Furthermore, when comparing the sensitivity across the six selected epitopes, DeepTCR outperformed and demonstrated the highest median sensitivity (∼0.35), followed by HD and GLIPH2 (∼0.15), whereas TCRdist3, TCRMatch, and LD had the lowest value (Fig. 5C). Overall, DeepTCR emerged as the most effective method for capturing epitope-specific sequences.

### Comparative robustness of clustering methods under increasing noise

**Figure 6.**
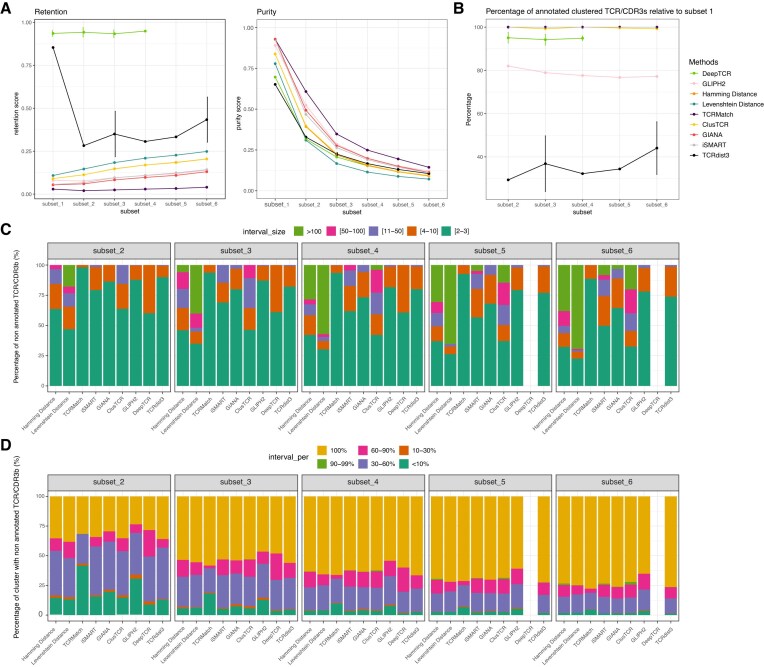
Clustering behavior of methods in the presence of noise. Comparison among the nine methods using 2876 unique TCR/CDR3bs sequences from 44 epitopes (Donor 1). (A) and (B) The *x*-axis shows six subsets: subset_1 (no spike-in) and subsets_2–6 (increasing amounts of irrelevant TCRβ/CDR3β sequences randomly spiked in annotated = binding score > 10, irrelevant/unannotated = <10). (**A**) Left: Retention; Right: Purity (definitions in the “Materials and methods” section), for each method accross subsets. (**B**) Persistence of annotated sequences: fraction of annotated, clustered sequences in each noisy subset (2–6) that were also clustered in subset_1 (no noise), assessing whether added noise displaces previously clustered annotated sequences. Point/bars show the mean over three runs; error bars indicate standard deviation. (**C**) Cluster size distribution of non-annotated sequences in noisy subsets (2–6) for each method, shown as the percentage of non-annotated TCRβ/CDR3β sequences assigned to clusters of size 2–3, 4–10, 11–50, 50–100, or >100. (**D**) Composition of clusters with respect to non-annotated sequences in noisy subsets (2–6), shown as the percentage of clusters containing <10%, 10%–30%, 30%–60%, 60%–90%, 90%–99%, or 100% non-annotated sequences.

To validate our previous observations, we analyzed a single-cell dataset from 10x Genomics generated using Feature Barcode technology (see the “Materials and methods” section). We assessed the clustering behavior of the different clustering methods in detecting either full TCRs or CDR3s (see Table 1) labeled with antigen specificity in the presence of increasing noise, using the Donor 1 dataset. The first subset (subset 1) is composed exclusively of TCRβ/CDR3β with a binding score >10 with any of the dextramers, resulting in 2876 antigen-specific CDR3β sequences. The subsets 2, 3, 4, 5, and 6 were, respectively, composed of 7613, 12 350, 17 087, 21 824, and 26 561 TCRβ/CDR3β sequences, including the 2876 antigen-specific TCRβ/CDR3β sequences and non-specific TCRβ/CDR3β pairs (binding score <10) randomly spiked to form five different subsets of increasing size and noise levels; subset 6 includes all the TCRβ/CDR3β from Donor 1. The objective was to assess each method’s robustness in retaining labeled TCRβ/CDR3β sequences within pure clusters despite the presence of noise, considering that the spiked-in sequences represent irrelevant, non-antigen-specific TCRs. A total of three runs for each subset were performed. For DeepTCR, memory limitations on our virtual machine prevented the generation of results for subsets 5 and 6. Although setting an arbitrary clustering threshold would have allowed us to obtain these results, such an approach would compromise comparability with other tools and the results obtained for the other subsets, for which cluster optimization is inherently determined by the algorithm. Clustering behavior was evaluated based on cluster retention and purity (Fig. 6A) as well as the percentage of antigen-specific TCRs clustered in the absence of noise (Fig. 6B). Among the tested methods, only DeepTCR and TCRdist3 showed variability in both metrics, as reflected by the standard deviations shown in Fig. 6A and B. The variability appears linked to their internal hierarchical clustering auto-optimization, which can sometimes yield a slightly different number of clusters.

**Table 1.** Comparative summary of method characteristics

Methods	Retention	Purity	% clusters with purity >0, 9	Cluster types	Scenario
clusTCR	Very low	Very high	Very high	Monospecific	1
GLIPH2	Low	Very high	Middle	Monospecific	1
TCRMatch	Very low	Very high	High	Mono-/polyspecific	1 and 2
GIANA	Low	High	Low	Mono-/polyspecific	2
iSMART	Very low	High	Middle	Mono-/polyspecific	2
TCRdist3	Middle	Middle	Very low	Mono-/polyspecific	2 and 3
DeepTCR	Very high	High	Low	Polyspecific	3
HD	Low	High	Low	Mono-/polyspecific	4
LD	Low	Middle	Low	Mono-/polyspecific	4

This table provides a systematic summary of the various characteristics of all the methods evaluated in the study. It categorizes each method’s clustering behavior across different metrics into qualitative intervals for ease of interpretation. These intervals are defined as follows: “Very Low” for values in the range of [0–0.2), “Low” for [0.2–0.4), “Middle” for [0.4–0.6), “High” for [0.6–0.8), and “Very High” for [0.8–1]. The fifth column summarizes the preference of cluster types over the six epitopes (Supplementary Table S5). The final column indicates the biological scenarios for which each method is most appropriate: (1) strict monospecific clustering with minimal cross-reactivity, (2) balanced trade-off between specificity and inclusiveness, (3) broad exploration of related (fuzzy) TCR groups, and (4) use of simple metrics for rapid broad grouping. Together, these categorizations provide a practical framework to guide method selection in relation to specific analytical objectives.

For most of the methods, except DeepTCR and TCRdist3, retention scores were low (in average 10% of the subset 1) and slightly increase by at most a two-fold for the largest subset (10 times larger than the subset 1) (Fig. 6A, left panel). DeepTCR exhibited a high and stable retention score regardless of the increased number of irrelevant sequences. TCRdist3, which showed a high retention score in the absence of noise, displayed a surprising drop with the addition of irrelevant sequences on subset 2, with no additional impact by increasing the noise (subsets 3–6). Nevertheless, regardless of the retention rate, all the methods exhibited a major decrease in purity with the addition of irrelevant sequences (Fig. 6A, right panel). This was not due to a loss of the antigen-specific sequences, as most if not all the antigen-specific sequences retained without noise were kept in the presence of increasingly irrelevant TCRs (Fig. 6B), except for TCRdist3 (and to a lesser extent GLIPH2). This could suggest either that the irrelevant sequences clustered together or that they contaminated antigen-specific clusters. By analysing the size of the clusters in which the irrelevant sequences were retained (Fig. 6C and Supplementary Fig. S7) and their distribution into the clusters (Fig. 6D and Supplementary Fig. S8), we found that most of the irrelevant sequences were forming small clusters between themselves. Altogether, these analyses revealed that while most of the methods retain sequences regardless of their relevance in terms of antigen specificity, they all seemed to mostly maintain antigen-specific sequences separated from irrelevant ones.

## Discussion

Given the exponential increase in the generation of TCR sequences and their use for clinical studies or novel therapeutics development, it is now of utmost importance to be able to determine their antigen specificity. Following significant progress in the field of bioinformatics, many tools have been developed by various teams to infer TCR specificity using different strategies such as theoretical frameworks, computational algorithms, and deep learning approaches. However, none of these methods were compared using a standardized dataset allowing them to evaluate their clustering behaviors. In this study, we conducted an independent comparison of the inference capabilities of nine widely used methods.

To accurately and robustly address this benchmark, we developed a curated and unified database ensuring reliable assignment of specificity to TCR sequences was reliable. To this end, we established a harmonized database from three public databases that, while enriched manually by authors of articles addressing this topic, are prone to errors [19–21]. We implemented an internal scoring system to assess the reliability of specificity assignments and selectively included TCRs with high-quality annotations. Additionally, we manually corrected the database to remove detectable inconsistencies, such as mismatches introduced during manual data entry by original sources. This forms a usable resource that will require continuous update with the novel entry of additional sequences as time goes. Indeed, the dynamic nature of TCR specificity databases such as VDJdb and IEDB is an important factor to consider when discussing both reproducibility and the interpretation of these results. In this study, we used a frozen snapshot of these databases collected in March 2023, and all analyses were performed on this version. To evaluate the potential impact of database evolution, we compared the content of the versions used in our analysis with their most recent releases (VDJdb: 21 February 2025; IEDB: 25 March 2025). For VDJdb, ~15 000 new sequences (TRA or TRB) have been added, ∼4800 removed, and ∼68 700 remained unchanged. For IEDB, ∼4000 sequences (TRA/TRB paired and unpaired) had been added, 37 removed, and ∼204 000 remained conserved. Beyond numerical changes, we also observed updates to annotation details, including additional metadata and corrections to prior entries. While these findings confirm that the databases are actively evolving, most entries used in our benchmarking remain present in the latest versions, underscoring the relevance of our results.

Using this unified database, we observed that methods such as LD, HD, TCRMatch, GIANA, and iSMART, which do not integrate paired TRA/TRB information, as well as clusTCR and GLIPH2, which only partially integrate TRA/TRB pairing, achieved lower retention and higher purity compared with methods that fully integrate TRA/TRB pairs. We also noted similarities between certain methods in terms of retention and cluster structure. Specifically, HD, LD (simple distance metrics not tailored to TCR sequences), and clusTCR displayed low retention and a non-negligible proportion of medium to large clusters (>5–10 sequences), exceeding 12% (Supplementary Table S3). These methods do not account for the physicochemical properties of amino acids, which may influence sequence connectivity patterns. Furthermore, under noisy conditions, HD and clusTCR produced remarkably similar results in both purity and retention. This resemblance is likely explained by the fact that clusTCR employs a similarity measure closely related to HD for cluster formation [9]. Another point to highlight is heterogeneity in cluster size across methods. For instance, GLIPH2 tends to generate relatively large clusters, with nearly 25% containing more than five sequences, whereas DeepTCR produces predominantly small clusters with 88.9% containing fewer than three sequences (Supplementary Tables S2 and S3). This contrast likely reflects fundamental differences in their underlying algorithms: GLIPH2 explicitly identifies sequence motifs, which can facilitate the grouping of multiple related CDR3s into the same cluster [16], while DeepTCR uses deep learning to extract implicit, discriminative “motifs”, and, in our analysis, applied its own hierarchical optimization, which may further promote the formation of small, highly specific clusters [17].

Regarding antigen specificity, methods based on simple distance measurements with a threshold of one, such as LD or HD, or relying on sequence alignment, including iSMART or GIANA, tended to form both polyspecific and monospecific clusters. In contrast, approaches based on k-mers such as TCRMatch or motifs such as GLIPH2 tend to generate monospecific clusters, as well as clusTCR. In contrast, TCRdist3 predominantly forms polyspecific clusters. A similar trend was observed with deep learning-based methods, such as DeepTCR, which also tended to form polyspecific clusters but achieved higher purity scores than TCRdist3. Polyspecificity refers to the ability of a single TCR to recognize distinct and unrelated epitopes, a well-established feature of TCR–pMHC interactions [27, 37]. For instance, the TCR composed of the CDR3α sequence CATDTTSGTYKYIF and the CDR3β sequence CSARDLTSGANNEQFF is annotated in the human pooled database as specific for multiple unrelated and distinct peptides, including ENPVVHFFKNIVTP (from myelin basic protein, *Homo sapiens*), IIPAFHFLKSEKGL (from calpain-7 like protein, *Homo sapiens*) or DVSKVHFFKGNGQT (from transporter atp-binding protein, *Rhizobium leguminosarum*) [38]. Furthermore, the specificity of a TCR can vary significantly when a TRB is paired with a different TRA [27]. Moreover, TCRs with completely dissimilar amino acid sequences may bind to the same epitopes. For example, in the pooled database, the CASSLLGGWSEAFF (CDR3b)–CAASHIQGAQKLVF (CDR3a) TCR and the CASSIRSSYEQYF (CDR3b)–CAAGGSQGNLIF (CDR3a) TCR bind both the GILGFVFTL epitope [39]. To be more specific, 12954 distinct TCRs are known to bind the GILGFVFTL epitope. Importantly, the mere binding of a TCR to an epitope does not guarantee T-cell activation [40]. Given that polyspecificity is a natural and very common phenomenon [6], having access to larger datasets of TCR pairs experimentally validated for their polyspecificity should help improve the methods and models behind. This has been recently started with the study of Messemaker *et al.* [41] and should help in better validating tools and their application.

Our analysis revealed a noticeable difference in metric outcomes when methods do not utilize paired TRA/TRB chains. Specifically, TRA chain sequences were more frequently organized into clusters than their TRB counterparts (Fig. 3D). This likely reflects the shorter average length of CDR3a sequences [16], [17], which increases their probability of being similar to one another, given that clustering is primarily driven by sequence similarity. This observation aligns with the established view that the TRB chain, with its greater diversity generated by VDJ recombination, generally plays a more dominant role in defining antigen specificity. In our comparative analysis, this property of TRB partly explains why it was the primary focus for benchmarking, as its higher diversity and stronger specificity signal may offer a more stringent test for clustering algorithms. Interestingly, the TRA chain clusters typically tended to be more polyspecific compared to those formed from TRB sequences. A previous study of the VDJdb identified overrepresentation of dual TRA expressing cells, determining whether the highly clustering TCRs belongs to that category would shed light on this particular phenomenon and the associated immune response [42]. This point is particularly important given that some methods in our study cluster TCRs by considering both the TRA and TRB chains, while others integrate only beta chains. Notably, even though tools like clusTCR, TCRdist3, and GLIPH2 theoretically have the capability to process data from paired TCRs, they are not optimized to perform paired chain clustering. Indeed, GLIPH2 assigns clusters based on the CDR3b region only, clusTCR generates an encoding for TRA and TRB sequences individually and then merges them, and TCRdist3 calculates a distance matrix for each chain and then adds them together. To determine the effectiveness of incorporating both TRA and TRB chain information, a detailed examination across these models, including DeepTCR, is necessary. This could highlight the unique strengths of each method, particularly valuable in the analysis of complex single-cell sequencing data.

When evaluating the clustering behavior of each method for six epitopes selected from different species and expressed differently in the filtered pooled database, we observed that TCRMatch and TCRdist3 demonstrated variability in their clustering outcomes forming both monospecific and polyspecific. Nevertheless, this selection enabled us a valuable perspective on how each method adapts to the epitope’s representation within the dataset. Furthermore, our findings indicate that DeepTCR exhibits superior sensitivity across six shortlisted epitopes, adeptly identifying sequences sharing the same specificity. Such polyspecificity or TCR promiscuity poses a particular challenge in clustering evaluation: biologically meaningful cross-reactivity may be misinterpreted as a loss of purity, especially when database annotations are incomplete or imprecise. Data quality issues, including misannotations and incomplete epitope labels, further complicate the interpretation of clustering behavior and highlight the importance of critically contextualizing these results.

Finally, the analysis using the 10x Genomics dataset provided confidence on the assumptions that TCRs with closely related sequences recognize similar antigens. Indeed, most methods tend to retain sequences irrespective of their antigen specificity but generally preserve a separation between relevant and irrelevant sequences. This suggests a certain robustness in preventing contamination of antigen-specific clusters, even if the overall retention values are inflated by irrelevant sequences. Methods like DeepTCR, with stable retention, and TCRdist3, despite its initial sensitivity to noise, illustrate different trade-offs between specificity and stability that are linked to their underlying clustering strategies. For DeepTCR, this near-complete, though not perfect, retention of antigen-specific sequences is consistent with its very high retention score, suggesting that such a score should be interpreted considering its tendency to preserve sequences regardless of specificity, as reflected by its frequent generation of polyspecific clusters. For TCRdist3, the relatively low fraction of retained antigen-specific sequences should be interpreted considering the concomitant drop in retention under noise. The antigen-specific proportion closely mirrors the retention curve, indicating that the antigen-specific sequences observed clustered in noisy subsets were essentially those already clustered in subset 1 (without noise).

### Limitations of the study and perspectives

One major limitation of this study is the biased composition of public TCR databases. Indeed, as we have shown, these databases are currently mainly composed by TCR sequences from CD8+ T cells, specific for viral antigens and restricted to common HLA alleles such as HLA-A*02:01. It is still unclear how this may influence clustering generalization, especially for methods sensitive to features like V/J gene usage or other elements correlated with HLA background. Indeed, it is known that the T-cell subsets (e.g. CD4 versus CD8) and HLA background influence gene usage and somehow CDR3 amino acid composition [43–45]. While this impact may be reduced for tools that focus solely on CDR3, its extent remains to be formally assessed. Similarly, methods originally trained or tuned on a specific antigen class (e.g. DeepTCR on cancer data) may perform differently across datasets. However, in that case, it is more related to the dataset than to the reality of clustering. Moreover, public databases are also biased toward public TCRs (i.e. sequences that are recurrent across individuals), which limit their ability to capture the true diversity of the TCR repertoire. The overall distribution of TCR diversity across antigens (e.g. viral versus cancer versus bacterial) remains largely unknown. Altogether, to improve tools and approaches, a more diverse and representative dataset is necessary to evaluate how clustering methods perform across different contexts or classes of antigens.

Another limitation is that all methods benchmarked in this study either exclusively focus on or attribute significant importance to the CDR3 regions. While both CDR3α and CDR3β contribute to binding, in proportions that vary with the epitope and MHC, it has been shown that incorporating full-length chain information has improved predictive accuracy [18]. However, this is likely due to the limited availability of paired, full-length sequences in public datasets. Future benchmarks and tools could integrate broader sequence features as data coverage improves.

In line with this, most of the methods studied in this manuscript are sequence-based. As such, the representation of AA sequences, while potentially increased with physicochemical properties and/or biological features, does not fully encapsulate the entirety of relevant information. Structure-based specificity inference models considering the three-dimensional architecture of the TCR–peptide/MHC complex would be instrumental for determining both binding between the two entities [46]. Recent studies posited that hybrid models combining sequence- and structure-based methods could enhance performance and yield more accurate specificity predictions [47]. Nevertheless, the lack of three-dimensional structural data in public repositories presents a significant obstacle. To address this gap, tools like TCRmodel2 have emerged, using AlphaFold [48, 49] to predict structures directly from amino acid sequences, thus offering a practical solution to overcome this challenge [50]. Interestingly, other methods, such as TCR-Pred, use molecular structure based on the two-dimensional architecture of CDR3 sequences to predict TCR specificity [51]. However, the integration of structural predictions into routine TCR specificity inference remains relatively recent, and broad experimental validation and systematic benchmarking are still needed to establish their robustness across diverse datasets.

In a different line of concept, we purposedly used default parameters provided by the authors of each tool when applying each method to the different datasets, except for TCRdist3. These defaults are generally recommended as the most appropriate for a wide range of datasets and represent the conditions under which the tools are most often applied in practice. Nevertheless, parameter settings can substantially influence clustering behavior, and optimal results often require fine-tuning for the dataset of interest. For tools that allowed it, we applied their internal optimization procedures, notably for hierarchical clustering, to ensure that the clustering step was adapted to the data, while still relying on the authors’ recommended configuration. Our aim was therefore to evaluate each method under conditions that reflect its intended general use, while allowing built-in optimizations where possible, to maintain both fairness and practical relevance in the comparison. Additionally, complementary metrics, such as pairwise F1 score computing to all labeled TCR–TCR pairs and balancing precision and recall, could provide further insight into clustering behavior. Although we did not include this metric in the present analysis, we consider it as a valuable addition for future benchmarking.

Finally, using the 10x Genomics datasets provided additional insights into the capacity of the different methods to actually provide biologically relevant sequence clustering. Nevertheless, it has to be noted that when cells are incubated with a mixture of dextramers, several peptide-MHC complexes may compete to bind to TCRs expressed on the cell surface. This competition can skew binding scores, particularly for TCRs with intermediate affinity for several dextramers. A highly abundant dextramer or one with high affinity can “monopolize” binding, masking other potential interactions. Moreover, the chosen threshold applied on the binding score for detecting specific TCRs based on 10x Genomics recommendations has been proposed to maximize detection specificity and reduce false positives. However, it can also increase false negatives notably for TCR with low-affinity or weakly expressed TCRs may have been excluded from the analysis.

### Conclusions and practical recommendations

Taken together, our study and results provide a comparative view of the strengths and limitations of current sequence-based approaches, while our curated TCR dataset offers a reliable resource for method evaluation. This benchmarking highlights that no single TCR clustering method is universally optimal, and that tool selection should be guided by the intended biological question. Based on our results, we propose several scenarios to help users select the suitable method in Table 1: Scenario 1: For applications requiring strictly monospecific clusters with minimal cross-reactivity and non-conservative approaches such as clusTCR, TCRMatch, and GLIPH2 are particularly suited. These methods consistently produce highly pure clusters, although GLIPH2 tends to form larger clusters than clusTCR and TCRMatch. Scenario 2: When a balance between specificity and inclusiveness is desired, iSMART, GIANA, and TCRdist3 represent intermediate options, generating clusters of moderate size with high purity, GIANA offering slightly higher retention than iSMART. TCRMatch produces very pure and generally smaller clusters, while TCRdist3 offers high retention and broad grouping. Scenario 3: For broader exploration of related (fuzzy) TCR groups, conservative methods such as DeepTCR and TCRdist3 provide high retention and capture a wide diversity of related sequences, with DeepTCR generally producing smaller clusters than TCRdist3. TCRdist3 also offers high retention and broad grouping but produces a mixture of mono- and polyspecific clusters. Scenario 4: Simpler distance metrics such as HD and LD may also be employed for broad grouping, though they exhibit lower retention. In certain contexts, a two-step strategy may be advantageous, beginning with a conservative method to delineate broad similarity groups, followed by a stricter approach to refine clusters and enhance specificity. These recommendations, together with the unified TCR database developed in this study, should provide a practical framework for selecting clustering methods aligned with distinct analytical objectives and should provide help in the interpretation when using tools for TCR specificity inference, supporting both fundamental immunology research and the development of antigen-specific therapies.

## Supplementary Material

lqaf150_Supplemental_Files

## Data Availability

The public databases, IEDB, McPAS, and VDJdb, were downloaded between January and March 2023 from the home page of their original websites. These databases and the final pooled database used in this analysis are available on Zenodo (https://doi.org/10.5281/zenodo.17240160). Information on how to download these data and the results of each clustering method is also stored in this url. All the scripts required for this study, i.e. public database and output processing, additional functions, etc., are supplied on Zenodo (https://doi.org/10.5281/zenodo.17240160), as are the data. The main benchmark script is available as a Rmarkdown file and reproduces all the figures presented in the document.
