# Supplementary material for: Benchmarking unsupervised methods for inferring TCR specificity
Source: NAR Genom Bioinform. 2025 Nov 19;7(4):lqaf150. doi: 10.1093/nargab/lqaf150 (PMC12629845; doi:10.1093/nargab/lqaf150)
Supplement: lqaf150_Supplemental_Files [file lqaf150_supplemental_files.zip › SuppFigure_1_legend_final.pdf]

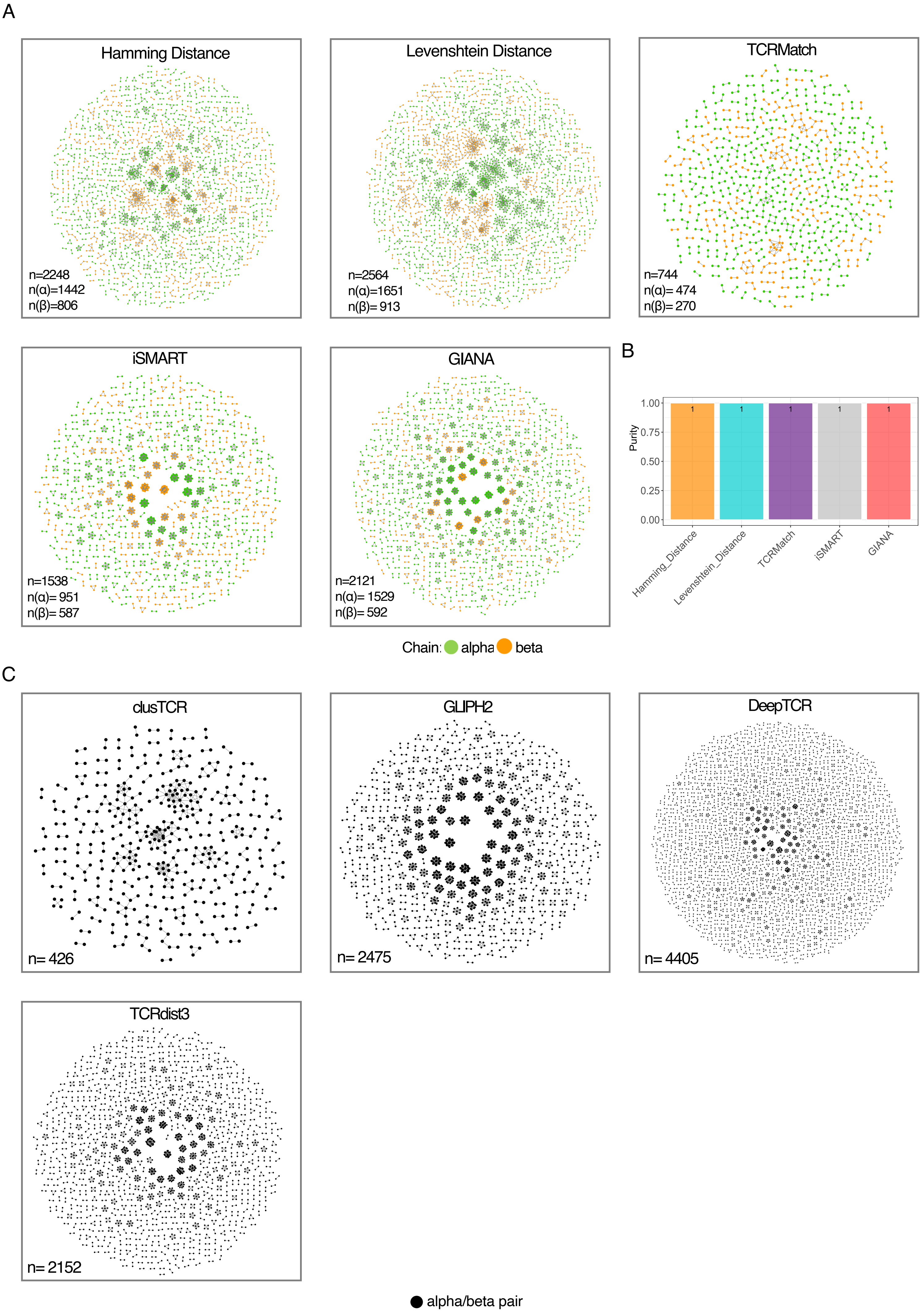

A
Hamming Distance
n=2248
n(α)=1442
n(β)=806
Levenshtein Distance
n=2564
n(α)=1651
n(β)= 913
TCRMatch
n=744
n(α)= 474
n(β)= 270
iSMART
n=1538
n(α)= 951
n(β)= 587
GIANA
n=2121
n(α)= 1529
n(β)= 592
Chain: alpha beta
B
Purity
1.00
0.75
0.50
0.25
0.00
1
1
1
1
1
Hamming_Distance
Levenshtein_Distance
TCRMatch
iSMART
GIANA
C
clusTCR
n= 426
GLIPH2
n= 2475
DeepTCR
n= 4405
TCRdist3
n= 2152
alpha/beta pair

Supplementary Figure 1: Network representations of clusters across methods with TRA/TRB information. (A) Network visualization of output clusters: this section displays networks generated by five methods - Hamming distance (HD), Levenshtein distance (LD), TCRMatch, iSMART and GIANA. Each subnetwork represents a cluster, with individual dots symbolizing CDR3 sequences; green denotes CDR3a and orange the CDR3b. The connectivity criteria vary for each method: HD=1 for HD (top-left panel), LD=1 for LD (top-middle panel), a TCRMatch score higher than 0.97 (top-right panel) and manual linkage of sequences within the same cluster for both iSMART (bottom-left panel) and GIANA (bottom-middle panel). (B) Purity analysis: this graph illustrates the purity of clusters generated by the five first methods according to the chain type. (C) Networks representing the output clusters for clusTCR, GLIPH2, DeepTCR and TCRdist3. A subnetwork is a cluster. A dot is an alpha/beta pair and it is colored in black. Network for clusTCR: two sequences are linked according the edgelist provided by the tool (HD=1). Network for GLIPH2: all sequences belonging to a cluster are manually linked together. Network for DeepTCR: all sequences belonging to a cluster are manually linked together. Nework for TCRdist3: a hierarchical clustering was performed on the TCRdist3 matrix and two sequences are manually linked together when belonging to the same cluster.
