# Supplementary material for: Benchmarking unsupervised methods for inferring TCR specificity
Source: NAR Genom Bioinform. 2025 Nov 19;7(4):lqaf150. doi: 10.1093/nargab/lqaf150 (PMC12629845; doi:10.1093/nargab/lqaf150)
Supplement: lqaf150_Supplemental_Files [file lqaf150_supplemental_files.zip › SuppFigure_2_revised.pdf]

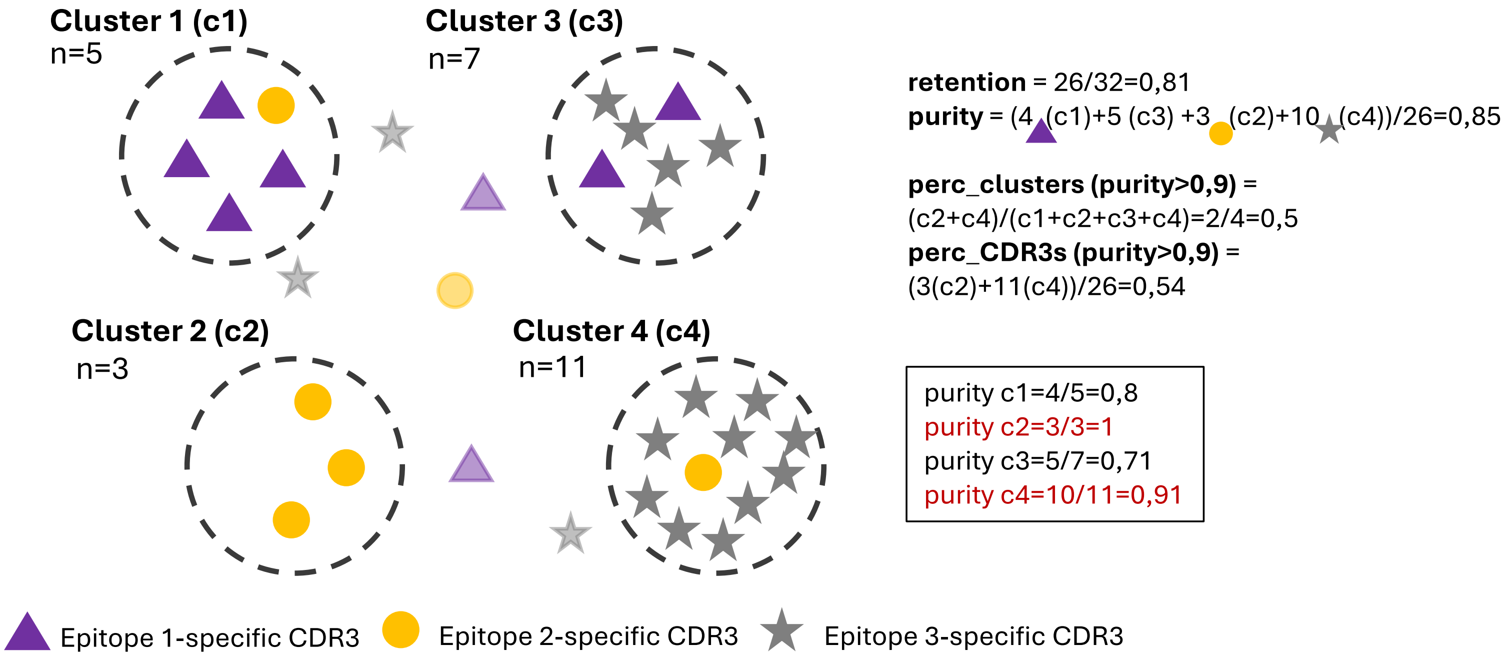

Supplementary Figure 2 : Methodology for metric calculation: Schema illustrating the process of calculating clustering evaluation metrics. It provides an example of possible clustering of CDR3 sequences known to bind three distinct epitopes (represented as stars, circles and triangles). Metrics have the following outcomes. Retention: this is the fraction of clustered sequences. In the given example, with 6 sequences outside any clusters, retention is 26/32=0.81. Purity: it assesses the fraction of CDR3s within a single cluster targeting the same epitope. Considering the largest epitope in each cluster, the sum divided by the total number of clustered sequences (4+5+3+10)/26 equals 0.85. Percentage of clusters with a purity over 0.9, individual purity is calculated for each cluster, but only those with a purity over 0.9 are considered. In this example, with individual purities of 0.8 (c1), 1 (c2), 0.71 (c3), and 0.91 (c4), only the c2 and c4 clusters are considered, resulting in 0.5. Percentage of sequences in the above-mentioned high-purity clusters yielding here 3 (c2) + 10 (c2) / 26 = 0.54.
