# Supplementary material for: Benchmarking unsupervised methods for inferring TCR specificity
Source: NAR Genom Bioinform. 2025 Nov 19;7(4):lqaf150. doi: 10.1093/nargab/lqaf150 (PMC12629845; doi:10.1093/nargab/lqaf150)
Supplement: lqaf150_Supplemental_Files [file lqaf150_supplemental_files.zip › SuppFigure_3_revised.pdf]

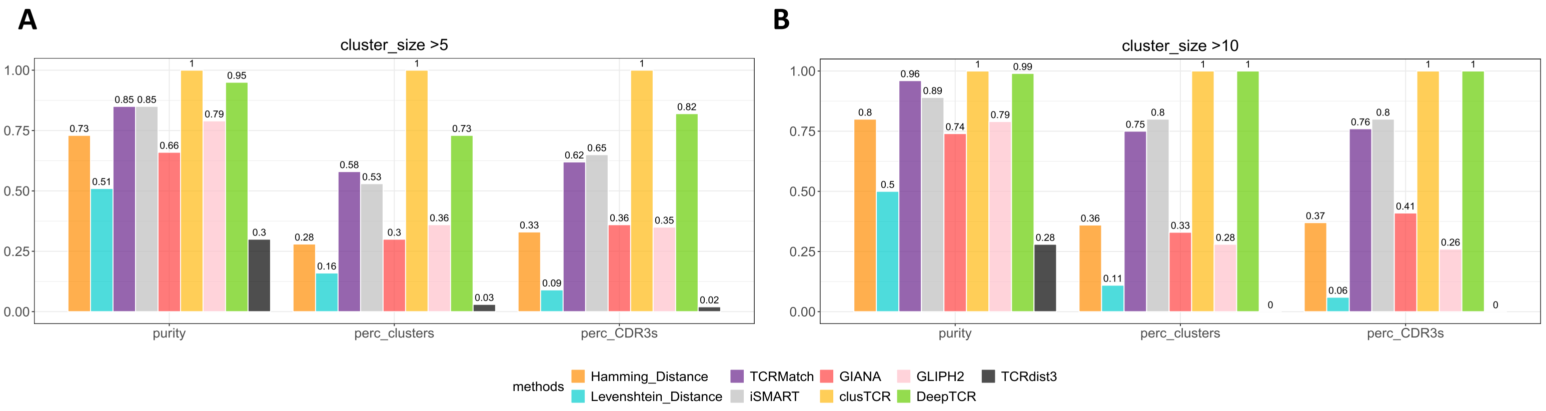

Supplementary Figure 3 : Detailed performance of methods. Barplots showing the performance evaluation metrics of the nine methods, as in the Figure 3.2.2, with a focus on varying cluster size thresholds: more than 5 and 10 sequences/pairs (A and B respectively).
