# Supplementary material for: Benchmarking unsupervised methods for inferring TCR specificity
Source: NAR Genom Bioinform. 2025 Nov 19;7(4):lqaf150. doi: 10.1093/nargab/lqaf150 (PMC12629845; doi:10.1093/nargab/lqaf150)
Supplement: lqaf150_Supplemental_Files [file lqaf150_supplemental_files.zip › SuppFigure_7_revised.pdf]

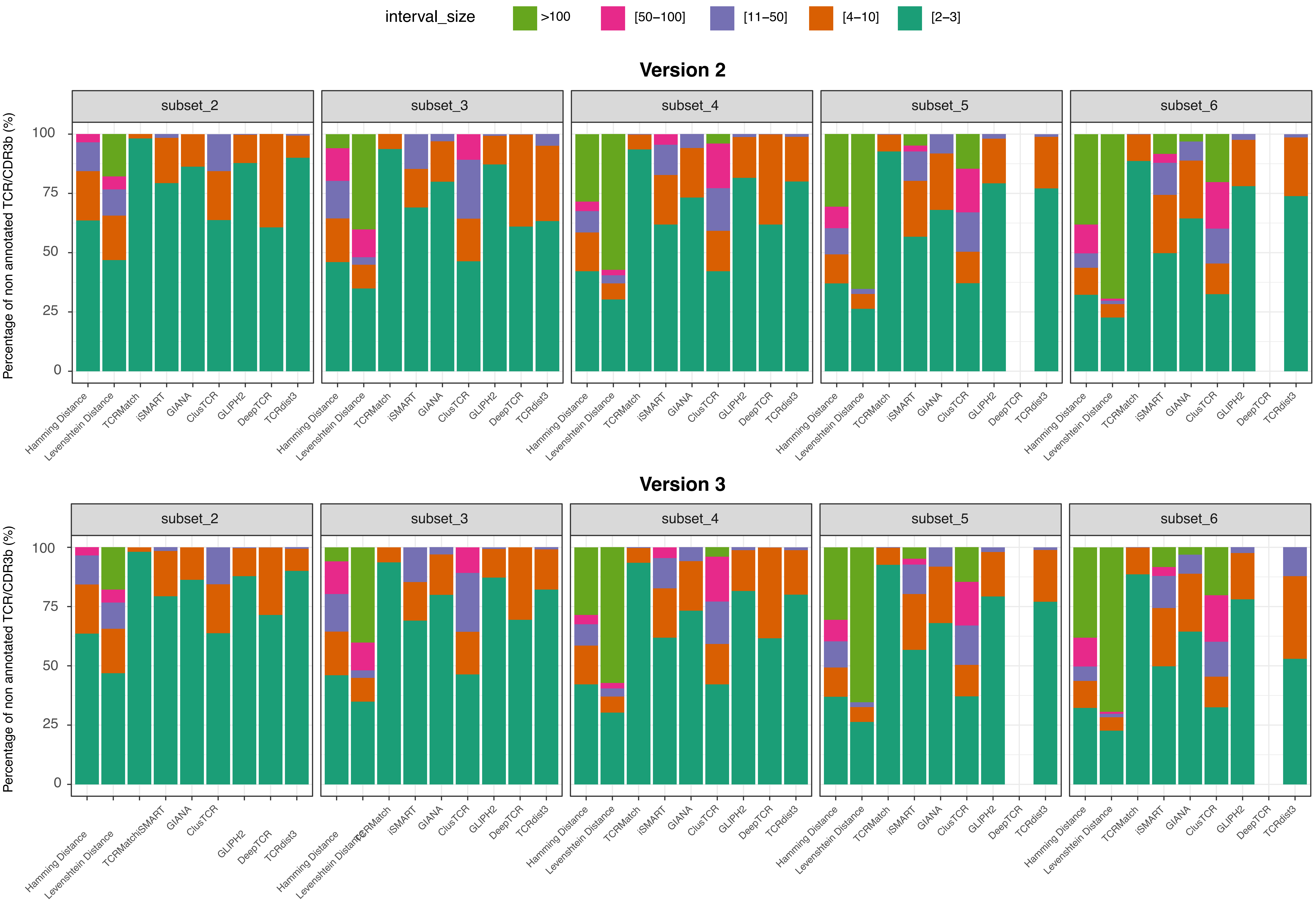

Supplementary Figure 7: **Cluster size distribution of non-annotated sequences in noisy subsets (2-6) for each method, shown as the percentage of non-annotated sequences assigned to clusters of size 2-3, 4-10, 11-50, 50-100 or >100.**
