# Supplementary material for: Benchmarking unsupervised methods for inferring TCR specificity
Source: NAR Genom Bioinform. 2025 Nov 19;7(4):lqaf150. doi: 10.1093/nargab/lqaf150 (PMC12629845; doi:10.1093/nargab/lqaf150)
Supplement: lqaf150_Supplemental_Files [file lqaf150_supplemental_files.zip › SuppFigure_8_revised.pdf]

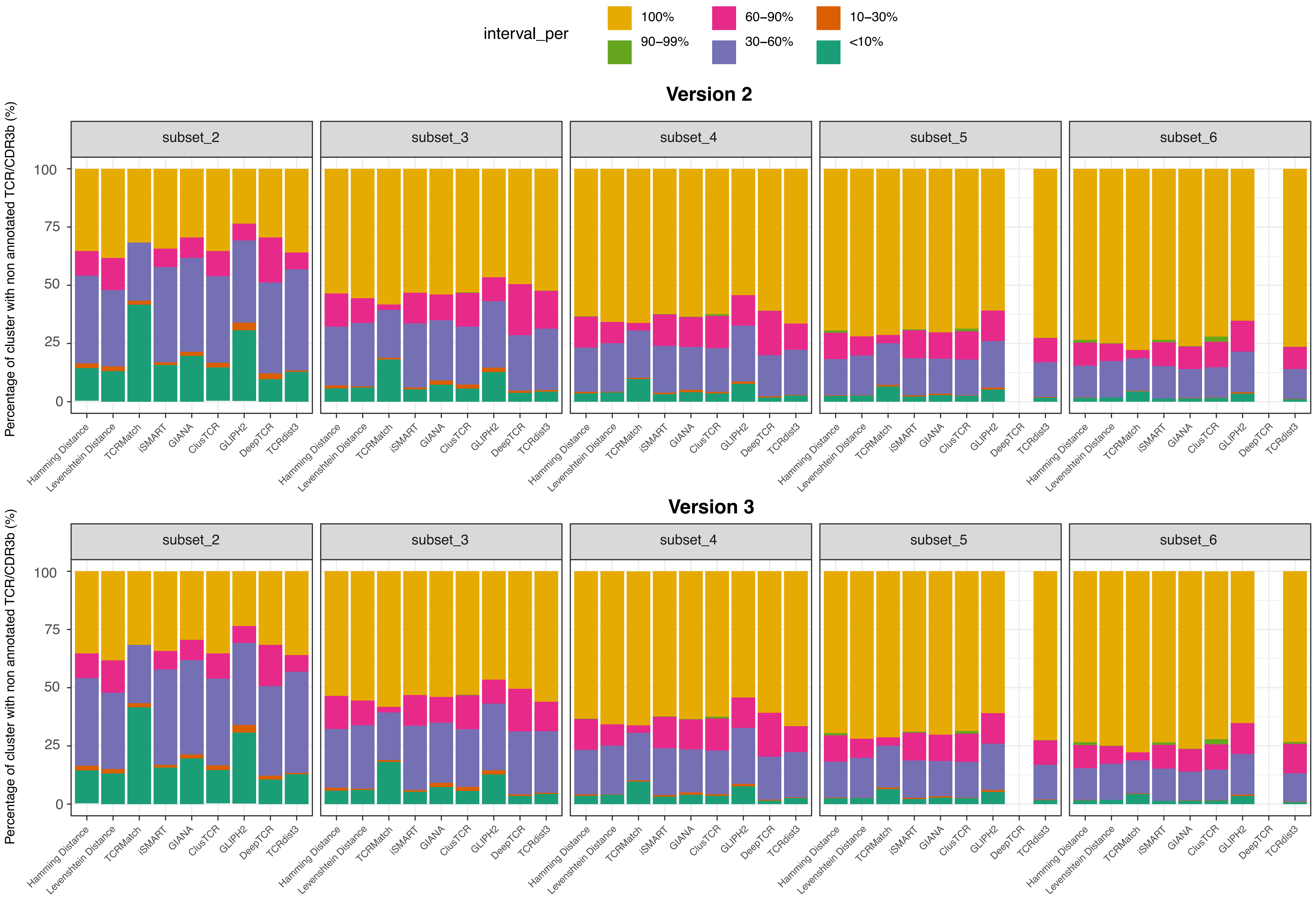

Supplementary Figure 8: Composition of clusters with respect to non-annotated sequences in noisy subsets (2-6), shown as the percentage of clusters containing <10%, 10-30%, 30-60%, 60-90%, 90-99%, or 100% non-annotated sequences
