# Supplementary material for: Benchmarking unsupervised methods for inferring TCR specificity
Source: NAR Genom Bioinform. 2025 Nov 19;7(4):lqaf150. doi: 10.1093/nargab/lqaf150 (PMC12629845; doi:10.1093/nargab/lqaf150)
Supplement: lqaf150_Supplemental_Files [file lqaf150_supplemental_files.zip › Supplemental_MatMeth_Tables_revised-clean.docx]

# *Supplemental Information*

Methods of interest

**- Similarity measures:**

**Levenshtein Distance (LD)** [1] and **Hamming Distance (HD)** [2]: both measure TCR sequence similarity, but LD accommodates sequences of varying lengths through edits (insertions, deletions, substitutions), while HD is limited to sequences of equal length, considering only substitutions. Commonly, sequences differing by one amino acid are deemed similar in both methods. From the CDR3 list, the LD and HD similarity matrices were calculated in R (using the stringdistmatrix() function in the stringdist package), filtered according to a threshold (HD=1, LD=1), then graphs were constructed (using the graph_from_data_frame, igraph package) identifying subnetworks as clusters.

**TCRMatch** (v.0.1.1). [3] Advances beyond LD and HD by using k-mer approach and the BLOSUM 62 matrix, specifically for TCR β-chain CDR3 (CDR3B) sequences [4]. It identifies and quantifies similarity with a final similarity score ranged from 0 to 1, with 1 indicating a perfect similarity. In our analysis, we set a high similarity threshold above 0.97. Similar R processing steps as HD and LD were applied to the filtered distance matrix for the identification of the final clusters. If the similarity threshold score decreases, the clusters will be much larger and the sequences within the cluster will be more diverse.

**TCRdist3** (v.0.2.2) [5] is the latest version of TCRdist [6] which offers a comprehensive analysis by including a range of CDR regions and taking into account paired αβ chains, applying a similarity-weighted mismatch distance. It uses the BLOSUM 62 matrix for scoring. For comparing TCRdist3 with the other methods, we applied a hierarchical clustering to its distance matrix as no current threshold exists as LD or HD. The optimal parameters for the clustering function (‘fcluster’ from the Python package ‘scipy’) were identified by maximizing the silhouette score of the clustering solution (not shown in the paper; threshold =118, optimal silhouette score =0.116).

- **Clustering methods:**

**ClusTCR** [7] uses k-means and hashing for speed, connecting similar sequences (HD = 1) within a network, followed by the Markov Clustering Algorithm (MCL) to identify dense substructures representing the clusters. The algorithm was run using the clusTCR Python package (v.1.0.2) <https://svalkiers.github.io/clusTCR/> using the CDR3α/β information and default parameters.

**iSMART** [8] group similar TCRs into antigen-specific clusters through pairwise local alignment using the BLOSUM62 matrix and taking into account the CDR3 sequence length. A depth-first search then identifies connected CDR3 clusters. iSMART was run using the Python script available in <https://github.com/s175573/iSMART> with default parameters and without V information.

**GIANA** (Geometric Isometry based TCR AligNment Algorithm, v.4.1) [9] transforms the CDR3 sequences into numeric vectors using multidimensional scaling (MDS) for fast nearest neighbour searches in the high-dimensional Euclidean space[9]. This method focuses on identifying pre clusters of CDR3 sequences, with additional filtering steps for final cluster formation. GIANA was run using the GIANA4.1.py script available in <https://github.com/s175573/GIANA> with default parameters and without V information.

**GLIPH2** [10] **is an update to GLIPH** [11] that clusters TCRs based on CDR3 similarity, using global and local similarity measures. It analyzes TCR sequences efficiently, overcoming the “small world effect” and employs the Fisher-exact test for cluster confidence, Gliph2 was run using the executable for Linux Centos available in [] with all default parameters provided by the authors. Files for CD8 reference, V usage and CDR3 length are contained in the human_v2.0 folder downloaded from the Gliph2 web site. In the column subjet:condition of the TCR input file, we have put the following information, “database:CD8+”.

- **DeepTCR** [12] (v.2.0) utilizes deep learning for complex TCR sequence analysis. It features a variational autoencoder for feature extraction from CDR3 sequences and offers multiple clustering methods like Phenograph , Hierarchical clustering, and DBSCAN. Hierarchical clustering is specifically used in this study for its efficiency in building nested clusters. For the reminder parameters, default ones provided by the DeepTCR Cluster() function were used for the clustering (i.e. linkage_method = “ward”, criterion = “distance”). As the distance threshold for the Hierarchical clustering was not provided, the function includes an automatic optimization of this value using the silhouette score. Furthermore, the 10X Genomics data from the pooled database were used as train data for DeepTCR (18 253 TCRα/β pairs among 42 unique epitopes).

| Antigen identification method | Type of antigen used | Antigen identification score |
| --- | --- | --- |
| Peptide-MHC (pMHC) Xmers sort | NA | 5 |
| In vitro stimulation in CD8 + T-cells | peptide | 4.5 |
|  | protein | 4.4 |
|  | pathogen | 4.3 |
|  | cell line | 4.2 |
|  | other | 4.1 |
| In vitro stimulation in CD4+ T-cells | peptide | 3.5 |
|  | protein | 3.4 |
|  | pathogen | 3.3 |
|  | cell line | 3.2 |
|  | other | 3.1 |
| Isolated from a specific tissue under a specific pathology / condition | NA | 2 |
| No verified | NA | 1 |
| Missing values | NA | 0 |

Supplementary Table 1 : Classification and scoring of antigen identification techniques

| Methods | Input information | Number of clusters (alpha – beta) | Number of small clusters (< 4 sequences) | Number of unique clustered sequences (CDR3a-CDR3b) or pairings |
| --- | --- | --- | --- | --- |
| Levenshtein Distance | CDR3a or CDR3b | 494 (299-195) | 370 (74.8%) | 2248 (1442-806) |
| Hamming Distance | CDR3a or CDR3b | 548 (361-187) | 409 (74.6%) | 2564 (1651-913) |
| TCRMatch | CDR3a or CDR3b | 285 (188-97) | 253 (88.8%) | 744 (474-270) |
| iSMART | CDR3a or CDR3b | 465 (285-180) | 372 (80%) | 1538 (951-587) |
| GIANA | CDR3a or CDR3b | 593 (417-175 +1) | 457 (77%) | 2121 (1529-592) |
| clusTCR | Paired CDR3a and CDR3b | **111** | 91 (82%) | 426 |
| GLIPH2 | Paired CDR3a and CDR3b + Vb + Jb + Count + Subject + Condition | 380 | **206 (54.2%)** | 1070 |
| DeepTCR | Paired CDR3a and CDR3b + Va + Ja + Vb + Jb + Count | **1628** | 1447 (88.9%) | 4405 |
| TCRdist3 | Paired CDR3a and CDR3b + Va + Ja + Vb + Jb + Count | 669 | 538 (80.4%) | 2093 |

Supplementary Table 2 : Description of required input information and numbers of unique clusters/sequences/pairings formed for each method using the unified database. Input information, number of clusters and number of small clusters (less than 4 sequences) are shown. To note, the numbers of alpha and beta clusters are displayed for methods which do not take into account pairings. Only GIANA has a heterogeneous cluster. Also, the number of unique clustered sequences and pairings are shown. Number of clustered CDR3a and CDR3b is shown for methods which do not consider pairings.

| **Methods** | **Nb of clusters (>3)** | **Nb of clusters (>5)** | **Nb of clusters (>10)** |
| --- | --- | --- | --- |
| Hamming Distance | 139 | 74 | 28 |
| Levenshtein Distance | 124 | 67 | 38 |
| TCRMatch | 32 | 12 | 4 |
| iSMART | 93 | 43 | 10 |
| GIANA | 136 | 66 | 21 |
| clusTCR | 20 | 14 | 6 |
| GLIPH2 | 174 | 94 | 46 |
| DeepTCR | 181 | 63 | 18 |
| TCRdist3 | 131 | 62 | 22 |

Supplementary Table 3 : Number of clusters formed for each method when considering clusters with size > 3, 5 or 10 sequences/pairs.

| Epitope | Species | Count unique CDR3beta | | Count unique CDR3alpha | Count unique CDR3alpha/CDR3beta |
| --- | --- | --- | --- | --- | --- |
| GILGFVFTL | Influenza | 456 | 484 | | 706 |
| NLVPMVATV | CMV | 349 | 325 | | 397 |
| GLCTLVAML | EBV | 170 | 145 | | 215 |
| FLCMKALLL | TAA | 136 | 134 | | 136 |
| NYNYLYRLF | SARS-CoV2 | 46 | 55 | | 56 |
| LLFGYPVYV | HTLV-1 | 7 | 4 | | 10 |

Supplementary Table 4 : Detailed overview of selected epitopes and associated species. CMV: Cytomegalovirus, EBV: Epstein Barr virus, TAA: Tumor associated antigen, SARS-Cov2: Severe acute respiratory syndrome coronavirus 2, HTLV-1: Human T-cell leukemia virus type I

| **Methods** | **GILGFVFTL** | **NLVPMVATV** | **GLCTLVAML** | **FLCMKALLL** | **NYNYLYRLF** | **LLFGYPVYV** | |
| --- | --- | --- | --- | --- | --- | --- | --- |
| **HD** | Mono/poly-specific | Polyspecific | Monospecific | Monospecific | Polyspecific | Mono/poly-specific |  |
| **LD** | Mono/poly-specific | Mono/poly-specific | Monospecific | Monospecific | Mono/poly-specific | Mono/poly-specific |  |
| **TCRMatch** | Monospecific | Mono/poly-specific | Monospecific | Monospecific | Monospecific | Polyspecific |  |
| **iSMART** | Mono/poly-specific | Polyspecific | Monospecific | Monospecific | Polyspecific | Mono/poly-specific |  |
| **GIANA** | Mono/poly-specific | Polyspecific | Monospecific | Monospecific | Polyspecific | Mono/poly-specific |  |
| **clusTCR** | Monospecific | Monospecific | Monospecific | X | Monospecific | Monospecific |  |
| **GLIPH2** | Monospecific | Monospecific | Monospecific | Monospecific | Monospecific | Monospecific |  |
| **DeepTCR** | Polyspecific | Polyspecific | Polyspecific | Polyspecific | Polyspecific | Polyspecific |  |
| **TCRdist3** | Polyspecific | Polyspecific | Polyspecific | Monospecific | Monospecific | Mono/poly-specific |  |

Supplementary Table 5 : Cluster types for the clustering of the 6 epitopes for each method. Barplots representing the percentage of clusters according to the number of specificities for NLVPMVATV, GLCTLVAML, NYNYLYRLF and LLFGYPVYV are not shown in this paper. There are no FLCMKALLL-specific clusters for clusTCR.

# References

[1] A. Madi *et al.*, « T cell receptor repertoires of mice and humans are clustered in similarity networks around conserved public CDR3 sequences », *eLife*, vol. 6, p. e22057, juill. 2017, doi: 10.7554/eLife.22057.

[2] B. Jabri *et al.*, « TCR Specificity Dictates CD94/NKG2A Expression by Human CTL », *Immunity*, vol. 17, n^o^ 4, p. 487‑499, oct. 2002, doi: 10.1016/S1074-7613(02)00427-2.

[3] W. D. Chronister *et al.*, « TCRMatch: Predicting T-Cell Receptor Specificity Based on Sequence Similarity to Previously Characterized Receptors », *Front. Immunol.*, vol. 12, mars 2021, doi: 10.3389/fimmu.2021.640725.

[4] S. Henikoff et J. G. Henikoff, « Amino acid substitution matrices from protein blocks », *PNAS*, vol. 89, n^o^ 22, p. 10915‑10919, nov. 1992, doi: 10.1073/pnas.89.22.10915.

[5] K. Mayer-Blackwell *et al.*, « TCR meta-clonotypes for biomarker discovery with tcrdist3 enabled identification of public, HLA-restricted clusters of SARS-CoV-2 TCRs », *eLife*, vol. 10, p. e68605, nov. 2021, doi: 10.7554/eLife.68605.

[6] P. Dash *et al.*, « Quantifiable predictive features define epitope-specific T cell receptor repertoires », *Nature*, vol. 547, n^o^ 7661, p. 89‑93, juill. 2017, doi: 10.1038/nature22383.

[7] S. Valkiers, M. V. Houcke, K. Laukens, et P. Meysman, « clusTCR: a Python interface for rapid clustering of large sets of CDR3 sequences », *bioRxiv*, p. 2021.02.22.432291, févr. 2021, doi: 10.1101/2021.02.22.432291.

[8] H. Zhang *et al.*, « Investigation of Antigen-Specific T-Cell Receptor Clusters in Human Cancers », *Clin Cancer Res*, vol. 26, n^o^ 6, p. 1359‑1371, mars 2020.

[9] H. Zhang, X. Zhan, et B. Li, « GIANA allows computationally-efficient TCR clustering and multi-disease repertoire classification by isometric transformation », *Nat Commun*, vol. 12, n^o^ 1, p. 4699, août 2021, doi: 10.1038/s41467-021-25006-7.

[10] H. Huang, C. Wang, F. Rubelt, T. J. Scriba, et M. M. Davis, « Analyzing the Mycobacterium tuberculosis immune response by T-cell receptor clustering with GLIPH2 and genome-wide antigen screening », *Nat Biotechnol*, vol. 38, n^o^ 10, p. 1194‑1202, oct. 2020, doi: 10.1038/s41587-020-0505-4.

[11] J. Glanville *et al.*, « Identifying specificity groups in the T cell receptor repertoire », *Nature*, vol. 547, n^o^ 7661, p. 94‑98, juill. 2017, doi: 10.1038/nature22976.

[12] J.-W. Sidhom, H. B. Larman, D. M. Pardoll, et A. S. Baras, « DeepTCR is a deep learning framework for revealing sequence concepts within T-cell repertoires », *Nat Commun*, vol. 12, n^o^ 1, p. 1605, mars 2021, doi: 10.1038/s41467-021-21879-w.
