# Supplementary material for: Benchmarking unsupervised methods for inferring TCR specificity
Source: NAR Genom Bioinform. 2025 Nov 19;7(4):lqaf150. doi: 10.1093/nargab/lqaf150 (PMC12629845; doi:10.1093/nargab/lqaf150)
Supplement: lqaf150_Supplemental_Files [file lqaf150_supplemental_files.zip › Supp_Figure_4_revised.pdf]

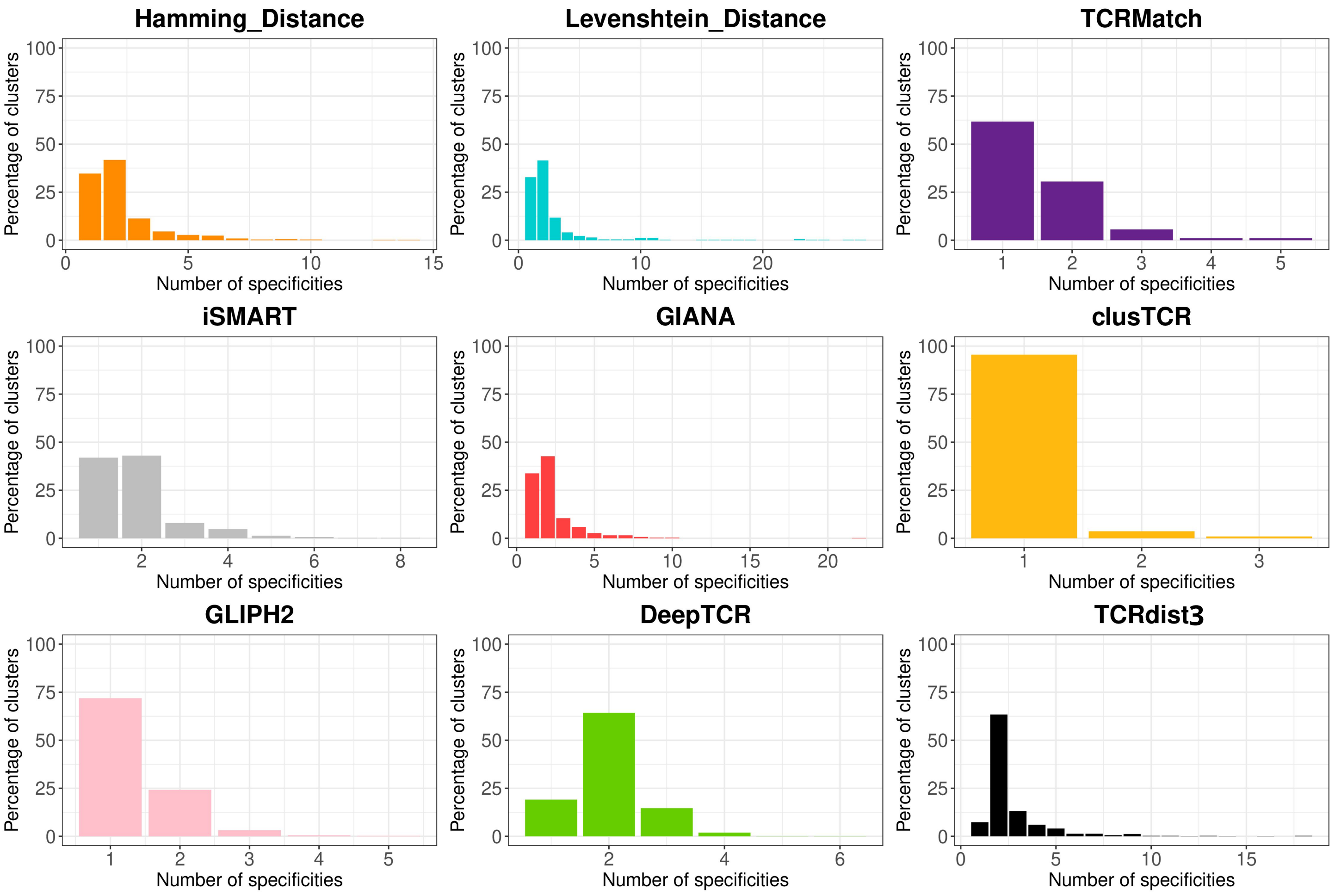

Supplementary Figure 4 : Cluster specificity distribution across methods. Percentage of clusters relative to the number of specificities contained within each cluster for each method.
