# Supplementary material for: Benchmarking unsupervised methods for inferring TCR specificity
Source: NAR Genom Bioinform. 2025 Nov 19;7(4):lqaf150. doi: 10.1093/nargab/lqaf150 (PMC12629845; doi:10.1093/nargab/lqaf150)
Supplement: lqaf150_Supplemental_Files [file lqaf150_supplemental_files.zip › Supp_Figure_5_revised.pdf]

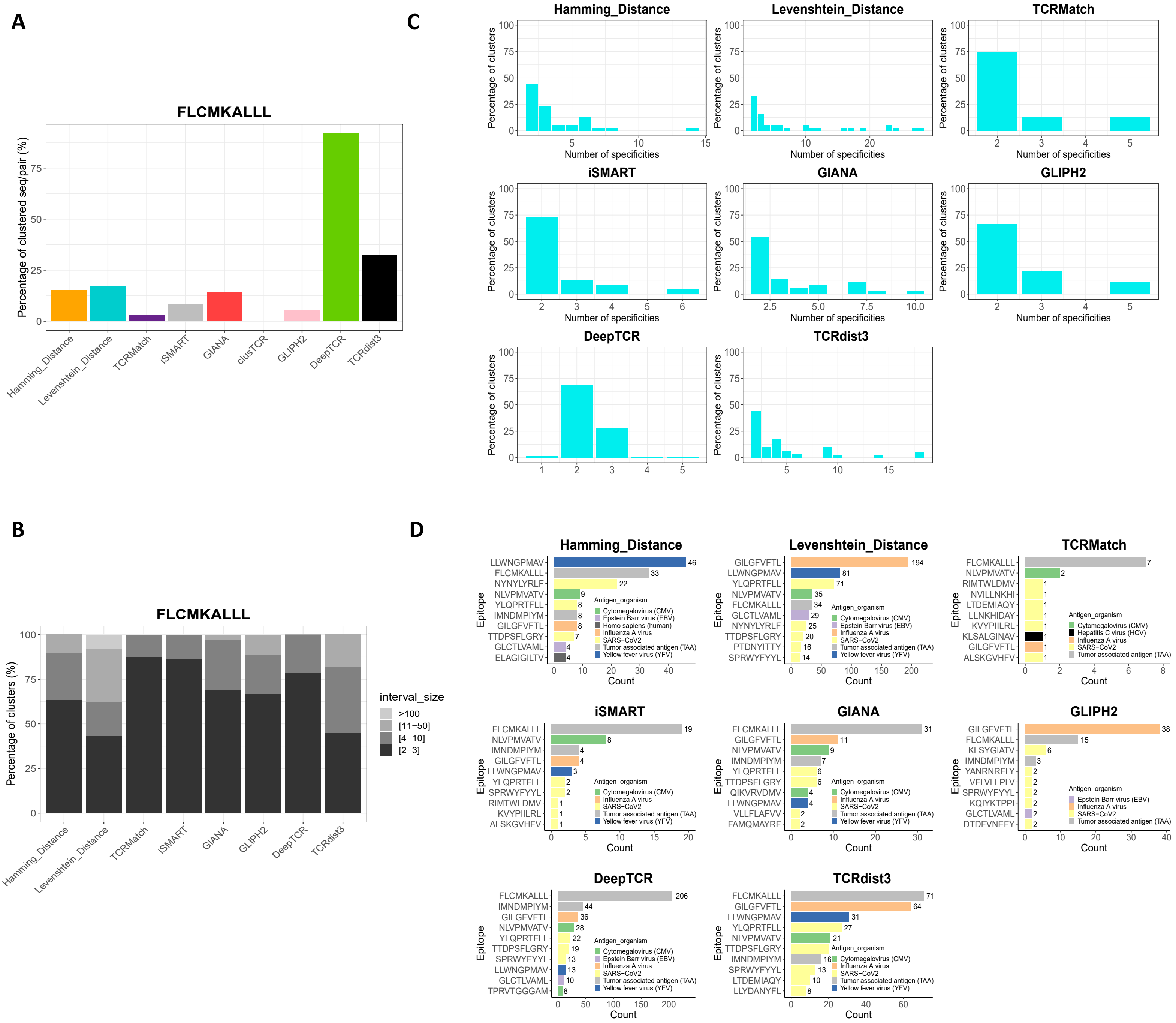

Supplementary Figure 5 : In-depth analysis of FLCMKALLL-specific-sequence/pair clustering. (A) Method-dependant clustering efficiency, displaying the percentage of FLCMKALLL-specific-sequences/pairs successfully clustered by each method. (B) FLCMKALLL-specific cluster size distribution for each method. Clusters with at least one specificity GILGFVFTL are considered as specific. (C) Specificity variation in clusters illustrating by the percentage of FLCMKALLL-specific-clusters relative to the number of specificities for each method. (D) Diversity of co-occurring epitopes, illustrated by the top 10 (or less) additional epitopes found within FLCMKALLL-specific clusters, for each clustering method. The epitopes are color-coded according to the antigen organism.
