# Supplementary material for: Benchmarking unsupervised methods for inferring TCR specificity
Source: NAR Genom Bioinform. 2025 Nov 19;7(4):lqaf150. doi: 10.1093/nargab/lqaf150 (PMC12629845; doi:10.1093/nargab/lqaf150)
Supplement: lqaf150_Supplemental_Files [file lqaf150_supplemental_files.zip › Supp_Figure_6_revised.pdf]

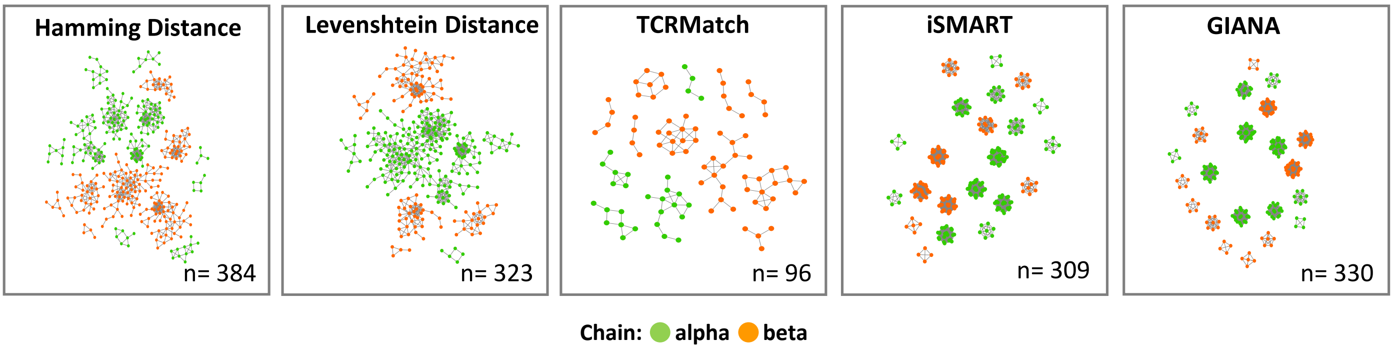


**Supplementary Figure 6:** Visualization of the most specific GILGFVFTL -specific clusters. Network representations of the purest GILGFVFTL-specific clusters (where GILGFVFTL is the major component, being at least twice as prevalent as the second most common epitope withi n the cluster), identified by the first five methods. Each network is color-coded by chain type (CDR3a in green CDR3b in orange).
